# Proteomic changes in Alzheimer’s disease associated with progressive Aβ plaque and tau tangle pathologies

**DOI:** 10.1038/s41593-024-01737-w

**Published:** 2024-08-26

**Authors:** Alexa Pichet Binette, Chris Gaiteri, Malin Wennström, Atul Kumar, Ines Hristovska, Nicola Spotorno, Gemma Salvadó, Olof Strandberg, Hansruedi Mathys, Li-Huei Tsai, Sebastian Palmqvist, Niklas Mattsson-Carlgren, Shorena Janelidze, Erik Stomrud, Jacob W. Vogel, Oskar Hansson

**Affiliations:** 1 Clinical Memory Research Unit, Department of Clinical Sciences Malmö, Lund University, Lund, Sweden; 2 Department of Psychiatry, SUNY Upstate Medical University, Syracuse, NY USA; 3 Rush University Alzheimer’s Disease Center, Rush University, Chicago, IL USA; 4 Cognitive Disorder Research Unit, Department of Clinical Sciences Malmö, Lund University, Malmö, Sweden; 5 Picower Institute for Learning and Memory, MIT, Cambridge, MA USA; 6 Department of Brain and Cognitive Sciences, MIT, Cambridge, MA USA; 7 University of Pittsburgh Brain Institute and Department of Neurobiology, University of Pittsburgh School of Medicine, Pittsburgh, PA USA; 8 Memory Clinic, Skåne University Hospital, Malmö, Sweden; 9 Department of Neurology, Skåne University Hospital, Lund, Sweden; 10 Wallenberg Center for Molecular Medicine, Lund University, Lund, Sweden; 11 Department of Clinical Sciences Malmö, SciLifeLab, Lund University, Lund, Sweden

**Keywords:** Alzheimer's disease, Alzheimer's disease

## Abstract

Proteomics can shed light on the dynamic and multifaceted alterations in neurodegenerative disorders like Alzheimer’s disease (AD). Combining radioligands measuring β-amyloid (Aβ) plaques and tau tangles with cerebrospinal fluid proteomics, we uncover molecular events mirroring different stages of AD pathology in living humans. We found 127 differentially abundant proteins (DAPs) across the AD spectrum. The strongest Aβ-related proteins were mainly expressed in glial cells and included SMOC1 and ITGAM. A dozen proteins linked to ATP metabolism and preferentially expressed in neurons were independently associated with tau tangle load and tau accumulation. Only 20% of the DAPs were also altered in other neurodegenerative diseases, underscoring AD’s distinct proteome. Two co-expression modules related, respectively, to protein metabolism and microglial immune response encompassed most DAPs, with opposing, staggered trajectories along the AD continuum. We unveil protein signatures associated with Aβ and tau proteinopathy in vivo, offering insights into complex neural responses and potential biomarkers and therapeutics targeting different disease stages.

## Abstract (web summary)

Using human cerebrospinal fluid proteomics, the authors found that proteins associated with Aβ pathology in Alzheimer disease were mainly expressed in glial cells, whereas those associated with tau tangle were linked to metabolism and mainly expressed in neurons.

## Main

AD pathology is characterized by the formation of insoluble protein aggregates, including plaques containing Aβ fibrils and neurofibrillary tangles containing tau fibrils^1,2^. Aβ plaques are the first pathology to accumulate in the brain, facilitating the accumulation and spread of tau tangles throughout the neocortex decade(s) later, at which point clear neurodegeneration and cognitive impairment ensue^3^. Although the canonical AD pathology is well known, it is increasingly clear that the disease etiology is multifactorial and biological features beyond Aβ and tau are critical in AD pathogenesis^4,5^. To gain more precise insight into AD pathophysiology, it is thus important to deepen our knowledge about which proteins and biological pathways are independently or conjointly associated with insoluble Aβ plaques and tau tangles. Such insight can lead to new biomarkers for disease staging or monitoring and, perhaps more critically, to identification of new disease mechanisms that might be targeted in future therapeutic interventions. Furthermore, although AD is the most common cause of dementia, many non-AD neurodegenerative diseases also lead to dementia and systematic proteomic comparisons between different conditions is important to dissect converging or diverging biological pathways leading to neurodegeneration across diseases^6^.

Proteomic studies on postmortem tissue have shown the benefit of increasing sample size into the hundreds to account for heterogeneity^7,8^. More recently, large in vivo cerebrospinal fluid (CSF) proteomic studies have also emerged. Such studies identified many DAPs between diagnostic groups in sporadic and autosomal dominant AD^6,9,10^, suggesting added value of proteomics to capture cognitive decline^11^, and highlighted common hits across proteomics in brain tissue and biofluids^12^. In most previous studies, AD pathology was measured with CSF Aβ and p-tau levels. However, such fluid biomarkers, especially p-tau, do not directly reflect pathology in the brain and the latter is more accurately captured with positron emission tomography (PET). In particular, tau-PET uniquely captures fibrillar tau tangle pathology, in contrast to soluble p-tau levels measured in CSF or plasma that reflect Aβ-plaque load rather than tau load^13,14^. Tau-PET is also more closely related to clinical progression and cognitive decline than fluid markers^15,16^, making it a key modality to study in relation to cellular and molecular changes. For these reasons, we combined CSF proteomics in a large sample covering the AD spectrum with PET radioligands measuring the loads of Aβ plaques and tau tangle pathologies, in cross-sectional and longitudinal analyses. Our main goal was to compare individuals based on their underlying AD pathology rather than their cognitive status, therefore capturing proteomic profiles specific to different pathophysiological stages of the disease, and to further characterize such proteins using imaging, transcriptomics and systems biology tools.

## Results

**Fig. 1:**
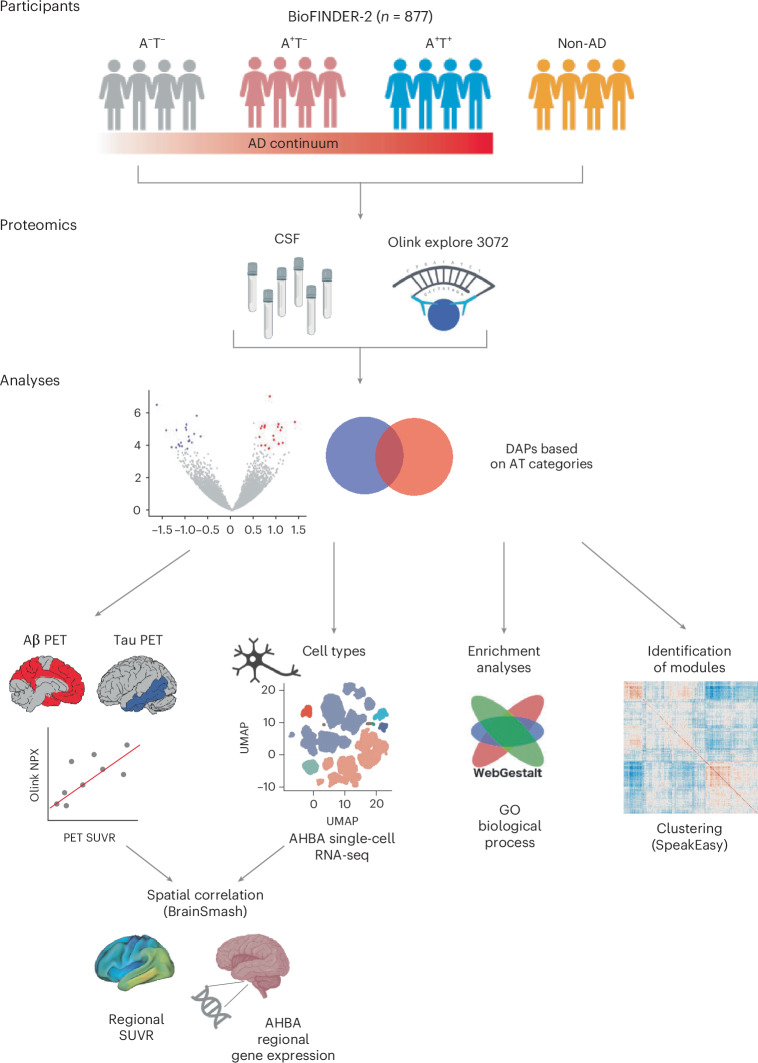
Study design overview. From all BioFINDER-2 participants with Olink proteomic data from CSF, we first assessed DAPs across the different A/T categories. From these DAPs, we then evaluated whether: (1) they were independently related to Aβ plaques or tau tangle pathology load (baseline PET) and rate of change (longitudinal PET); (2) the proteins’ regional gene expression in the brain matched the regional PET pattern; and (3) they were enriched in different cell types or biological processes using enrichment analyses. Last, we derived protein co-expression modules to investigate the overlap between such modules and the DAPs.

We studied 877 deeply phenotyped participants from the BioFINDER-2 cohort, in which thousands of CSF protein levels were analyzed (Olink Explore 3072 proximity extension assay). Individuals were grouped according to their positivity on Aβ and tau pathologies, assessed using the CSF Aβ42/Aβ40 ratio and tau-PET uptake in a temporal meta-region of interest (ROI), respectively (Methods). CSF Aβ42/Aβ40 ratio accurately detects the presence of Aβ plaques in the brain (A)^17^ and tau-PET detects insoluble tau fibrils in the cortex (T)^18,19^. CSF Aβ42/Aβ40 was chosen over Aβ-PET to create the A/T categories to maximize the sample size, because patients with dementia do not undergo Aβ-PET in the BioFINDER cohorts. Protein associations with continuous Aβ-PET standardized uptake value ratio (SUVR) are investigated in subsequent analyses. The A/T classification resulted in four groups of individuals: 352 A^−^T^−^ (Aβ and tau negative without a neurodegenerative disease diagnosis), 184 A^+^T^−^ (Aβ positive, tau negative), 231 A^+^T^+^ (Aβ and tau positive) and 110 non-AD (Aβ-negative patients with clinically diagnosed non-AD neurodegenerative diseases) (Table 1 and Extended Data Table 1). As expected, the A^+^T^−^ group and especially the A^+^T^+^ group contained more cognitively impaired individuals than the A^−^T^−^ group (Table 1). The first set of analyses focused on the AD spectrum (A^−^T^−^, A^+^T^−^ and A^+^T^+^) and the group of non-AD patients was investigated subsequently. Figure 1 displays an overview of the study design and main analyses.

**Table 1:** BioFINDER-2 cohort demographics

	A^−^T^−^ (*n* = 352)	A^+^T^−^ (*n* = 184)	A^+^T^+^ (*n* = 231)	Non-AD (*n* = 110)
**Age (years)**	63.65 ± 14.09	71.94 ± 8.16	72.75 ± 7.40	68.63 ± 9.57
**Sex, female** ***n*** **(% F)**	182 (52)	88 (48)	125 (54)	40 (36)
**Education (years)**	12.70 ± 3.34	12.33 ± 3.79	12.57 ± 4.41	11.88 ± 3.58
***APOE*ε4 carriers** ***n*** **(%)**	110 (31)	121 (66)	168 (73)	26 (24)
**MMSE**	28.60 ± 1.55	27.52 ± 2.49	22.73 ± 4.98	25.85 ± 3.69
**Cognitive status** **Unimpaired:MCI:Dementia**	262:90:0	94:70:20	22:66:143	See Extended Data Table 1
**CSF Aβ42/40 (NeuroToolKit)**^**a**^	0.11 ± 0.01	0.05 ± 0.01	0.04 ± 0.01	0.11 ± 0.01
**Aβ-PET SUVR (flutemetamol)**^**b**^	0.93 ± 0.08	1.29 ± 0.27	1.64 ± 0.23	−
**Tau-PET SUVR (RO948)**	1.14 ± 0.09	1.19 ± 0.09	2.1 ± 0.06	1.15 ± 0.11
**PET follow-up time (years)**^**c**^	2.65 ± 1.00	2.56 ± 0.97	1.90 ± 0.65	2.00 ± 0.39

Data are presented as mean ± s.d. unless specified otherwise. A: Aβ status (positive or negative) based on CSF Aβ42/Aβ40 ratio; T: tau status (positive or negative) based on tau-PET uptake in a temporal meta-ROI.

The non-AD group is composed of Aβ-negative patients with clinically diagnosed non-AD neurodegenerative diseases.

*APOE*ε4, apolipoprotein E genotype (carrying at least one ε4 allele).

^a^There are 8, 14, 23 and 5 missing values in the respective categories for which clinical assays were used to determine the Aβ status.

^b^There are 15, 26 and 141 missing values in the respective categories. Dementia patients do not undergo Aβ-PET, which explains the high missing frequency in A^+^T^+^ and the non-AD neurodegenerative categories.

^c^There are 65, 40, 87 and 53 individuals in the respective categories who did not have longitudinal data.

### Proteomic signatures of Aβ and tau pathology

**Fig. 2:**
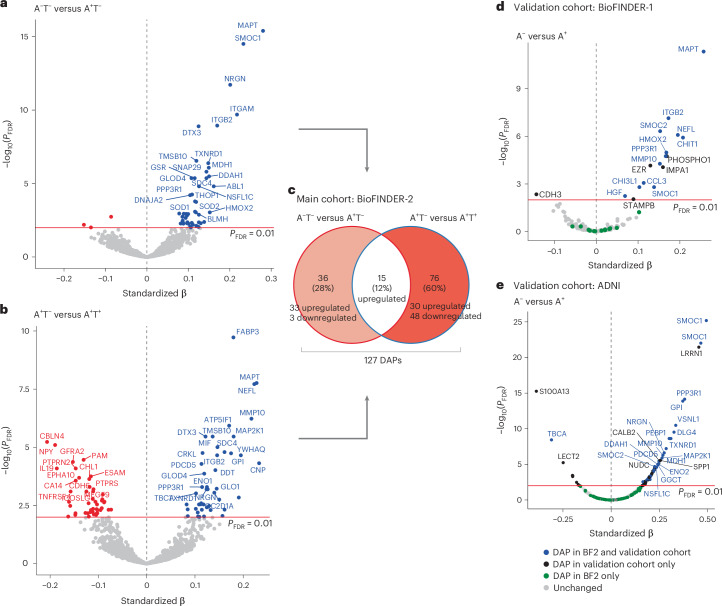
Differential protein abundance in CSF between AT comparisons. **a**,**b**, Volcano plots depicting DAPs in different groups: A^−^T^−^ (*n* = 352) versus A^+^T^−^ (*n* = 184) (**a**) and A+T^−^ versus A^+^T^+^ (*n* = 231) (**b**). Models included age, sex and mean overall protein level as covariates. The red line represents the threshold of *P*_FDR_ < 0.01, above which we considered proteins for subsequent analyses. Only the top proteins are labeled for legibility on the volcano plots. **c**, Venn diagram summarizing the comparisons shown in **a** and **b** based on proteins significant at *P*_FDR_ < 0.01 in the main cohort, BioFINDER-2. **d**, Volcano plots depicting DAPs in BioFINDER-1 (validation cohort, Olink proteomics) between A^−^ (*n* = 415) and A^+^ (*n* = 292) individuals, including age, sex and mean overall protein level as covariates. **e**, Volcano plots depicting DAPs in ADNI (validation cohort, SomaLogic proteomics) between A^−^ (*n* = 212) and A^+^ (*n* = 215), including age and sex as covariates. Some proteins appear twice given that different aptamers measured the same protein. A^−^ or A^+^ status was based on Aβ-PET. In **d** and **e**, the analysis was restricted to proteins that overlapped with those available in BioFINDER-2 (BF2). All standardized β values displayed come from two-sided linear regressions and all *P* values were adjusted for FDR.

We first assessed DAPs between individuals without elevated AD pathology compared with those positive for Aβ pathology only (A^−^T^−^ (no AD pathology) versus A^+^T^−^ (isolated Aβ pathology)). Subsequently, we compared the A^+^T^−^ group with individuals positive on both key AD pathologies (A^+^T^−^ versus A^+^T^+^ (both Aβ pathology and cortical tau tangle pathology)). These comparisons allowed investigation of protein differences along the expected pathological cascade of AD. All analyses included age, sex and mean overall protein level as covariates. Fifty-one proteins were differentially abundant (*P* value adjusted for false discovery rate (FDR) (*P*_FDR)_ < 0.01) between A^+^T^−^ and A^−^T^−^. Almost all of those (94%, 48 of 51) were upregulated in individuals with isolated Aβ pathology (Fig. 2a) and some of the main proteins included SMOC1, MDH1, SNAP29, SOD1 and SOD2.

Next, we studied which proteins changed with progression to cortical tau fibrillar pathology, that is, which proteins further differed when comparing A^+^T^−^ with A^+^T^+^ individuals. This comparison revealed more DAPs, with 45 upregulated proteins (48%) in individuals with both elevated Aβ plaque and tau tangle pathologies and 48 downregulated proteins (52%) (Fig. 2b). Overall, 15 proteins (CNP, CRKL, DTX3, GLOD4, ITGAM, ITGB2, MAP2K1, MAPT, MIF, NRGN, NSFL1C, PPP3R1, SDC4, TMSB10 and TXNRD) were commonly upregulated across the two comparisons, being elevated already in the A^+^T^−^ participants and showing further elevation in the A^+^T^+^ group (Fig. 2c). We hereafter refer to them as the ‘core proteins’, given that they were differentially abundant throughout the AD spectrum and increased with advancing AD pathology. DAPs specific to the A^+^T^−^ versus A^−^T^−^ contrast are subsequently referred to as ‘early DAPs’. Of those, SMOC1 showed the strongest effect and was elevated in A^+^T^−^ versus A^−^T^−^, but not further as fibrillar tau pathology develops (that is, when comparing A^+^T^+^ with A^+^T^−^). The proteins uniquely differentially abundant in A^+^T^+^ (against A^+^T^−^) were split into two categories of ‘late DAPs’: upregulated or downregulated late DAPs. Overall, 127 unique proteins were differentially abundant along the AD spectrum (Fig. 2c) and were retained for subsequent analyses. All summary statistics from the differential expression analyses are reported in Supplementary Table 1.

We validated a subset of the DAPs in an independent cohort of 631 participants from the BioFINDER-1 study (Extended Data Table 2), in which a smaller set of CSF proteins was quantified with Olink. From all proteins analyzed in BioFINDER-2, 202 overlapped in BioFINDER-1. In this validation cohort, we compared participants based on their Aβ status (213 A^+^ versus 418 A^−^) because tau-PET was unavailable (Fig. 2d and see Supplementary Table 2 for all statistical results). Overall, there was 92% consistency in differential abundance analyses between the two cohorts, with the strongest DAPs being found in both datasets. Similarly, we also replicated many of the main DAPs using 463 ADNI participants who had CSF proteomics quantified with SomaLogic (251 A^+^ and 212 A^−^). We analyzed SomaLogic proteins that matched the ones analyzed in BioFINDER-2, yielding an overall 90% consistency in significant DAPs between cohorts (Fig. 1e and Supplementary Table 3). SMOC1 stood out as the main protein more abundant in A^+^ compared with A^−^ in the AD Neuroimaging Initiative (ADNI), consistent with previous results.

**Extended Data Fig. 1:**
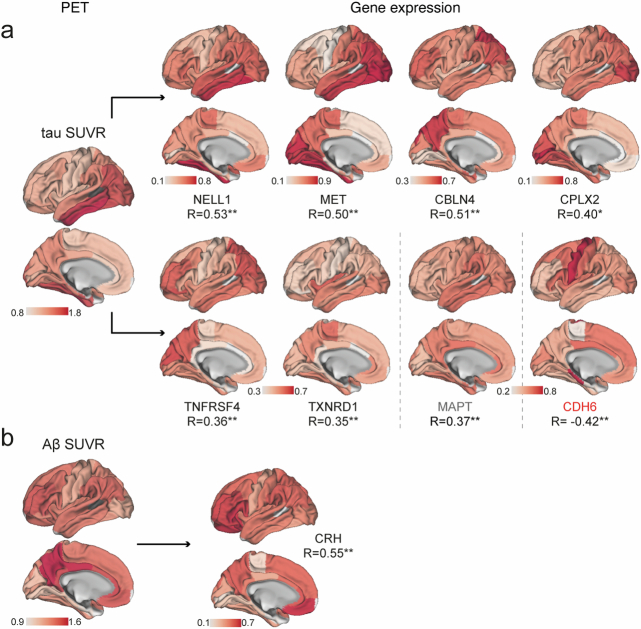
Correlations between regional gene expression and PET patterns. Differentially abundant proteins for which the regional gene expression (Allen Human Brain data) was related to either the regional tau- (**a**) or Aβ-PET (**b**) SUVR deposition. Only proteins significant after accounting for spatial autocorrelation and across two brain atlases (Desikan-Kiliany and Schaefer) are displayed. The only exception is MAPT (in gray), which was only significant in one atlas. CDH6 is in red as it is the only one with a negative correlation. Correlation coefficients and significance (two-sided test, no adjustment for multiple corrections) are shown from the Desikan-Kiliany atlas. See the Supplementary material for all detailed methods and results. * corresponds to p < 0.05, ** corresponds to p < 0.01.

In complementary analyses, we investigated whether the regional gene expression pattern of DAPs correlated with the pattern of Aβ or tau aggregates in the brain using imaging transcriptomics (Extended Data Fig. 1, Supplementary Table 4 and Supplementary Results). Overall, we found only moderate associations between regional RNA expression of seven DAPs and the tau-PET pattern and one with the Aβ-PET pattern.

### Distinct biological processes between early and late proteomes

**Fig. 3:**
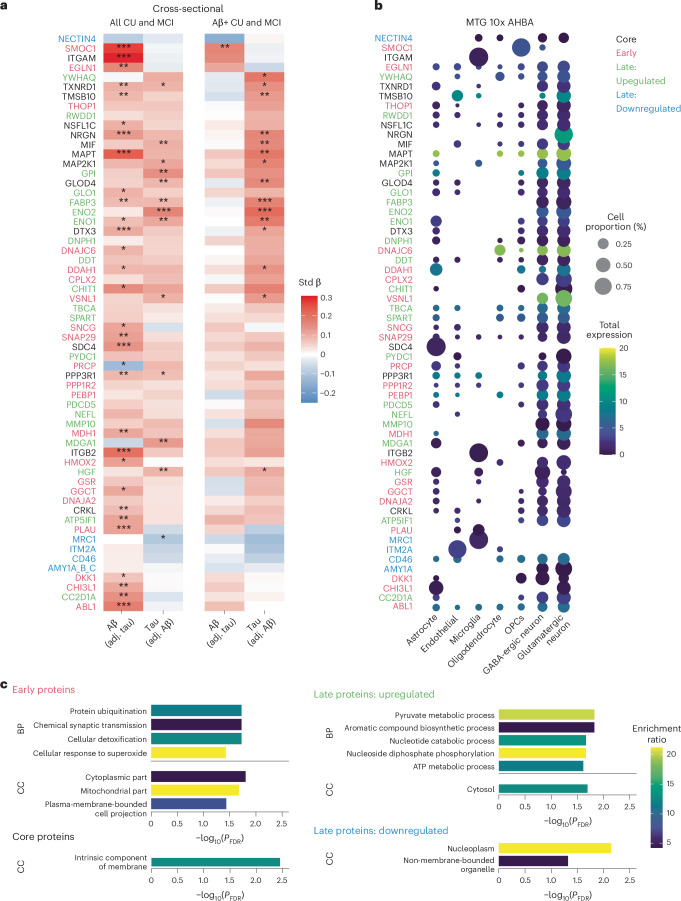
Characterization of DAPs with PET, cell type and functional enrichment. **a**, Standardized (Std) β coefficients from linear models relating AD fibrillar pathology (Aβ- and tau-PET SUVR both included as independent variables) to the CSF protein levels. Models included age, sex and mean overall protein level as covariates. Only proteins with significant associations are reported in the figure. **b**, Proportion of expression by cell type from single-cell transcriptomics data from the middle temporal gyrus for all proteins shown in **a**. To improve legibility, only average expressions >5% are displayed. **c**, Summary terms from functional enrichment analyses using GO databases from the different categories of proteins. For enrichment analyses the 1,331 Olink proteins were used as background. All linear regressions performed were two sided and *P* values were adjusted for FDR. ^*^P_FDR_ < 0.05, ^**^P_FDR_ < 0.01, ^***^P_FDR_ < 0.001. adj., adjusted.

**Extended Data Fig. 2:**
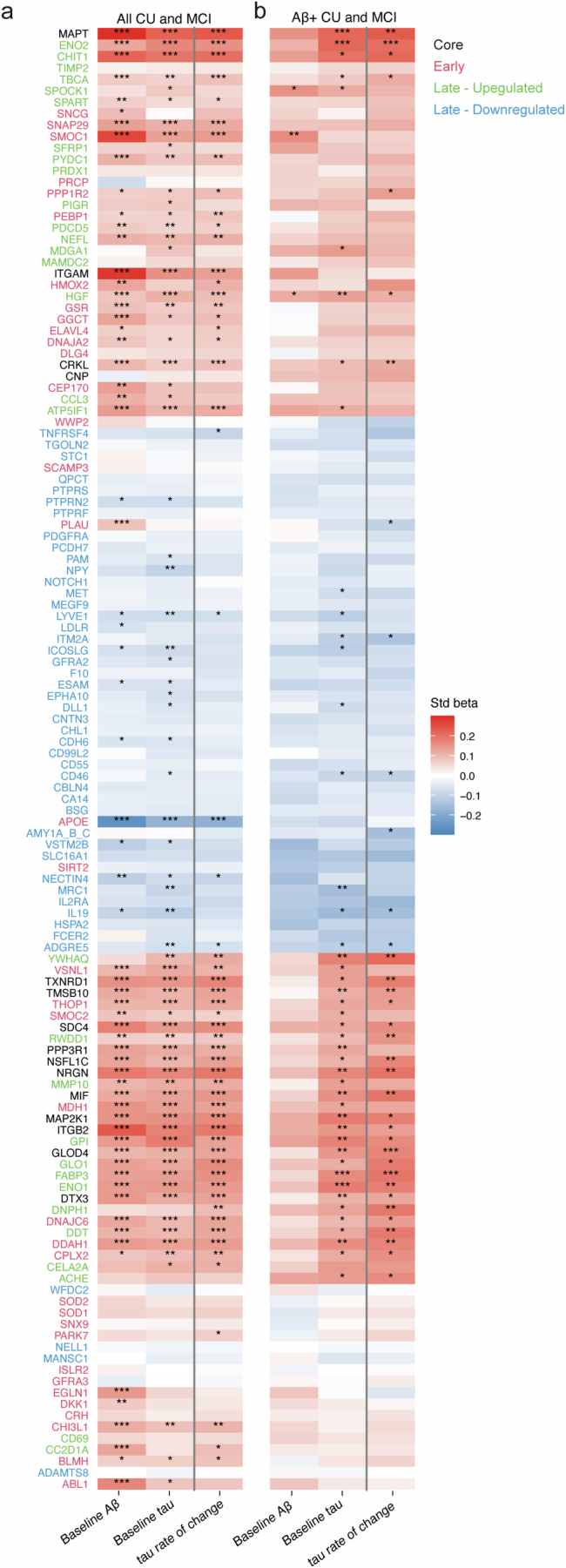
Aβ and tau PET association in separate models. **a, b**, Standardized beta coefficients from linear models relating AD fibrillar pathology at baseline (Aβ- and tau-PET SUVR included separately in different models) and over time (Aβ- and tau-PET rate of change included separately in different models) to the CSF protein levels of the 128 differentially expressed proteins. Models included age, sex, mean overall protein level as covariates. There was no significant associations with Aβ-PET rate of change, and thus those results are not displayed. Results in panel **a** included all CU and MCI participants. Results in panel **b** were restricted to Aβ-positive CU and MCI participants. All linear regressions performed were two-sided, and p-values were adjusted for false discovery rate. * corresponds to p_FDR_ < 0.05, ** corresponds to p_FDR_ < 0.01, *** corresponds to p_FDR_ < 0.001 Aβ = beta-amyloid; CU = cognitively unimpaired; MCI = mild cognitive impairment.

We next investigated whether the DAPs were associated with the load of either insoluble Aβ-containing plaques or tau fibrillar aggregates (cross-sectional analyses, Fig. 3a) as well as the accumulation over time of the two proteinopathies (longitudinal analyses, see below). Across all cognitively unimpaired (CU) individuals and patients with mild cognitive impairment (MCI), SMOC1 and ITGAM showed the strongest positive associations with global Aβ-PET levels (Fig. 3a), all associations being independent of tau-PET levels. When restricting the sample to Aβ-positive individuals, SMOC1 was the only protein that remained associated with Aβ-PET uptake, independently of tau. Several core and late upregulated proteins were independently related to tau-PET load, with associations being even clearer in the Aβ-positive group, as expected because tau-PET uptake is elevated in Aβ-positive individuals. In particular, FABP3, ENO2, ENO1 (enolase 1 and 2), MAPT, NRGN (neurogranin), MIF, TMSB10 and GLOD4 showed the strongest associations with tau-PET load: higher levels of these proteins in the CSF were related to greater baseline tau fibrillar pathology independent of Aβ-PET load (all standardized coefficients between 0.13 and 0.21, *P*_FDR_ from 0.01 to <0.001; Fig. 3a). See Supplementary Table 5 for all statistical results across the 127 DAPs and Extended Data Fig. 2 for results when Aβ- and tau-PET are investigated individually (that is, separate regression models for each pathology).

**Extended Data Fig. 3:**
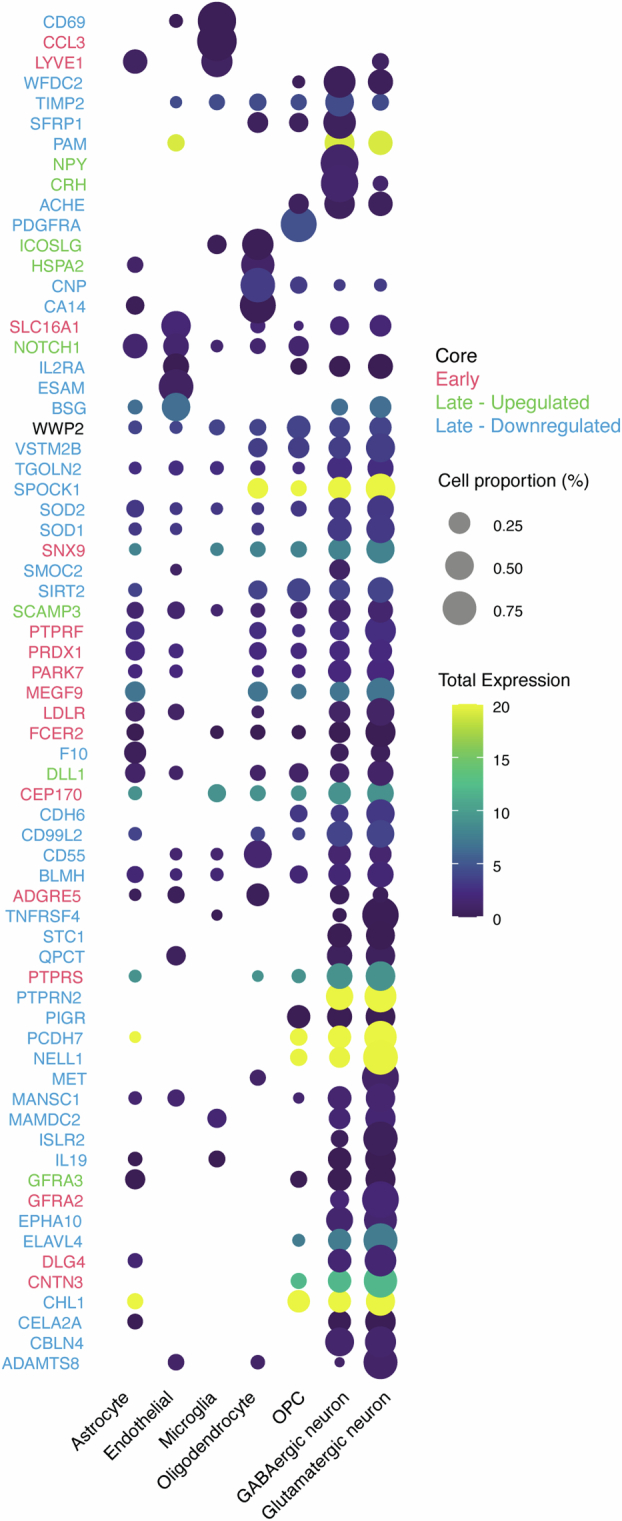
Average gene expression levels across cell types in the Allen Brain dataset. Proportion of gene expression by cell type from single-cell transcriptomics data from the middle temporal gyrus from the differentially expressed proteins that were not included in Fig. 3b. To improve legibility, only average expression above 5% are displayed. Transcriptomics data was downloaded from the reference donors of the Allen Brain Institute.

**Extended Data Fig. 4:**
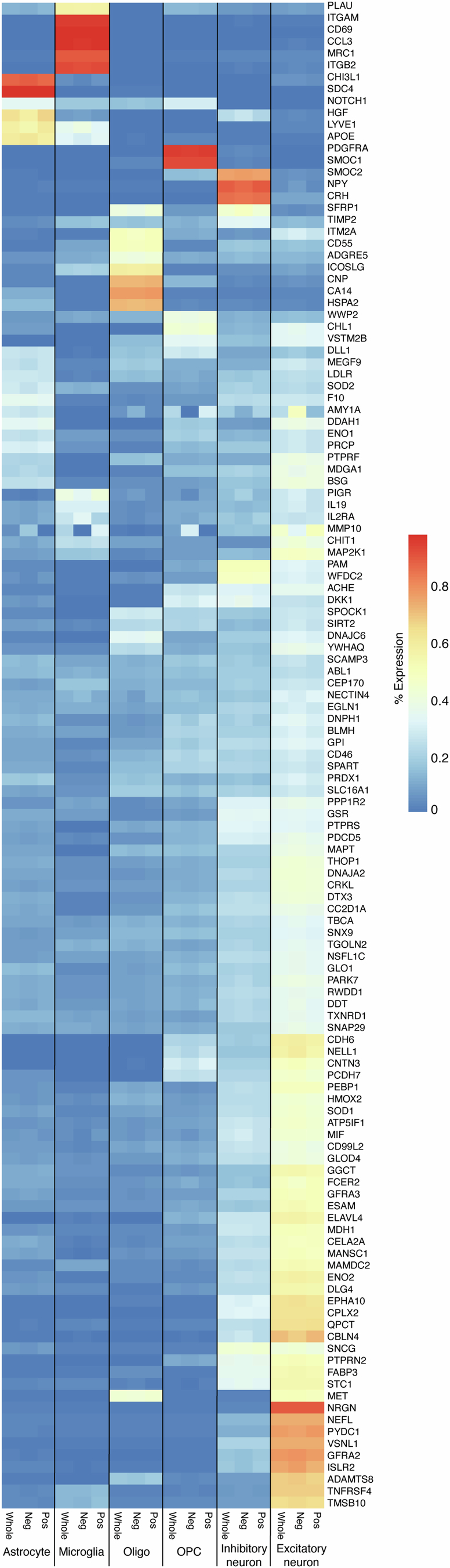
Average gene expression levels across cell types in the ROSMAP single nuclei data. Proportion of gene expression by cell type from single-nuclei data from the dorsolateral prefrontal gyrus of 427 ROSMAP donors. The 128 DAPs are depicted on the y-axis. Average expression was calculated across the whole sample (left column for each cell type, ‘Whole’), only A− donors (middle column for each cell type, ‘Neg’) and only A+ donors (right column for each cell type, ‘Pos’). The A− or A+ status was defined based on the CERAD score (A− being No AD or Possible; A+ being Definite or Probable). Transcriptomics data was accessed from Mathys et al, Cell, 2023.

**Extended Data Fig. 5:**
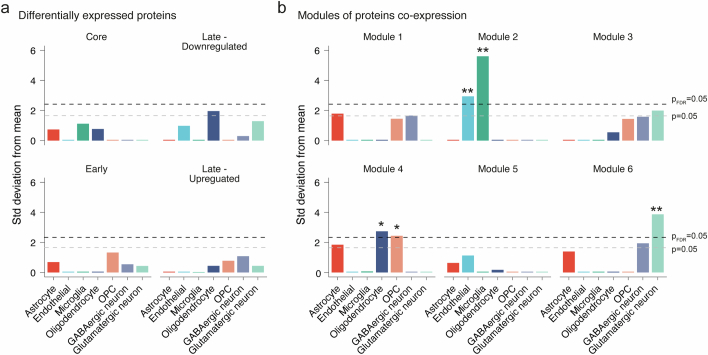
Cell-type enrichment analyses. **a, b**, Cell-type enrichment analyses based on single-cell transcriptomics data from the middle temporal gyrus based on the different categories of differentially expressed proteins (**a**) and taking all proteins part of the different biological modules (**b**). Bootstrap enrichment tests were performed, and all p-values were adjusted for false discovery rate. * corresponds to p_FDR_ < 0.05, ** corresponds to p_FDR_ < 0.01.

To gain greater insight into cell-type specificity of the DAPs, we calculated the percentage of messenger RNA expression of those proteins across different cell types, based on single-cell transcriptomics data (Allen Human Brain Atlas (AHBA); Fig. 3b and Extended Data Fig. 3). Most proteins were mainly expressed in neurons, split between glutamatergic and γ-aminobutyric acid (GABA)-ergic neurons. However, the few proteins strongly associated with levels of Aβ were expressed in different cell types: SMOC1 was the only protein specific to oligodendrocyte precursor cells (OPCs) and ITGAM and ITGB2 were among the few proteins specific to microglia. The same cell-type specificity was found when using a large single-cell transcriptomics dataset from 427 ROSMAP (Religious Orders Study/Memory and Aging Project) participants split into A^+^ and A^−^ instead, based on neuropathology (Extended Data Fig. 4)^20^. Comparing the neuronal versus glial expression of proteins associated with Aβ-PET versus the ones specifically associated with tau-PET (based on Fig. 3a), Aβ-related proteins were significantly more expressed in glial cells than the tau-related proteins, which were more neuronal (*χ*^2^ = 5.6, *P* = 0.03; 7 glial/10 neuronal proteins for Aβ; 0 glial/10 neuronal for tau). Quantitative cell-type enrichment analyses in the core, early, upregulated and downregulated groups of DAPs showed no significant enrichment of any cell type (Extended Data Fig. 5a). Functional enrichment analysis allowed further understanding of the biological roles of the DAPs (summary results in Fig. 3c; see Supplementary Table 6 for all significant gene ontology (GO) terms). Early proteins were enriched for terms related to synaptic transmission, cellular ubiquitination and detoxification and cellular response to free radicals, corresponding to different cellular components (mitochondria, cytoplasm and cell projection) (Fig. 3d). Upregulated late proteins were enriched for terms related to ATP process and glycolysis in the cytosol. Downregulated late proteins were enriched in cellular components related to nucleus and structures not bounded by lipid membrane (for example, ribosomes, cytoskeleton and chromosomes). In contrast, core proteins were enriched for terms related to the cellular membrane. In the more restricted set of proteins specifically associated with either Aβ- or tau-PET uptake (for example, from Fig. 3a), the tau-related DAPs were enriched for two biological processes similar to the late upregulated DAPs: NAD biosynthetic process and nucleotide phosphorylation (both *P*_FDR_ < 0.001), with the contributing proteins being ENO1, ENO2 and GPI (glucose-6-phosphate isomerase). The Aβ-related proteins had no significant GO enrichment.

**Extended Data Fig. 6:**
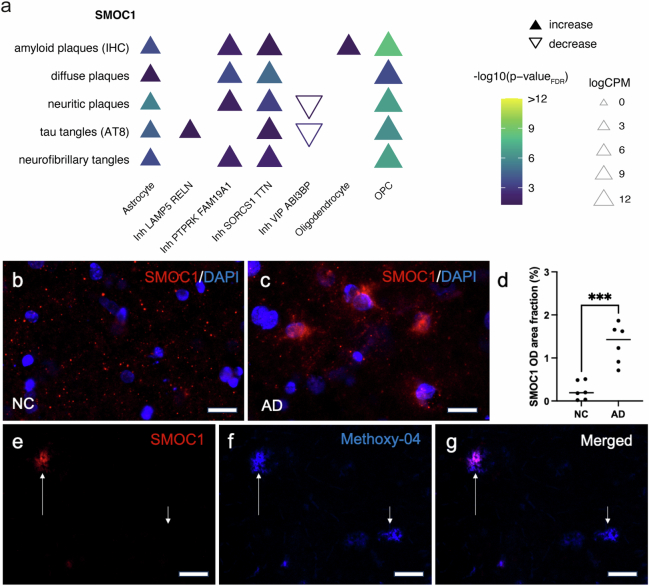
SMOC1 characterization in postmortem human tissue. **a**, Differential gene expression for SMOC1 (strongest protein associated with Aβ) across all cell types and with five measures of AD pathology was accessed from Mathys et al, *Cell*, 2023, based on single-nuclei data from 427 ROSMAP donors. Only associations (two-sided regressions) surviving correction for false discovery rate across all cell types are depicted. Amyloid plaque and tau tangle loads were quantified with immunohistochemistry (IHC) (AT8 antibody for tau) and averaged across 8 brain regions. Diffuse plaques, neuritic places and neurofibrillary were assessed with silver-stained slides in 5 brain regions. Overall there were higher SMOC1 levels with greater postmortem AD pathology predominantly in OPCs, and to some extent in astrocytes and some inhibitory neuronal subtypes, reflecting the cell types where this gene is mainly expressed. **b,c** Representative images of entorhinal cortex from a nondemented control (NC) (b) and an Alzheimer’s disease (AD) (c) patient stained against SMOC1 (red) and the nuclei marker DAPI (blue). **d**, Optical density (OD) area fraction of SMOC1 in entorhinal cortex in six NC and six AD patients. Each point represents the mean of the OD area fraction in six images captured from each case. The data was analyzed using 2-sided student t-test. Significant difference at ***p < 0.001. **e-g**, Example of double staining against SMOC1 (red) and Methoxy-04 staining plaques (blue). **e**, SMOC1 staining. **f**, Aβ plaques staining. **g**, Merged image of SMOC1 and plaques. SMOC1 were found in some (long arrow), but not all (short arrow) Methoxy-04 stained plaques, which were found in three out of six AD cases. Overall, we found higher SMOC1 levels in brain tissue from AD cases compared to controls, with SMOC1 localized in amyloid plaques. Scale bar in (b-c) = 10 μm and in (g) = 50μm.

Given the predominance of SMOC1 in the proteomic literature and that it showed the strongest associations with Aβ, we further characterized this protein using postmortem human data. We confirmed higher SMOC1 expression predominantly in OPCs with greater postmortem AD pathology and high SMOC1 levels in brain tissue from patients with AD (Extended Data Fig. 6).

### Select proteins presage subsequent tau-PET accumulation

**Fig. 4:**
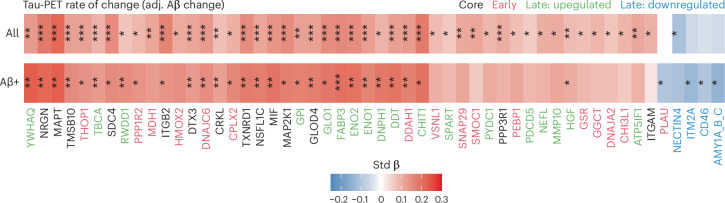
DAPs related to longitudinal tau-PET. Standardized β coefficients from linear models relating accumulation of AD fibrillar pathology (Aβ- and tau-PET rate of change both included as independent variables) to the CSF protein levels. Models included age, sex and mean overall protein level as covariates. Only proteins with significant associations with tau-PET rate of change are displayed, because there were no associations with Aβ-PET rate of change. The top row included all CU and MCI participants and the bottom row only Aβ-positive CU and MCI participants (as in Fig. 3a). All linear regressions performed were two sided and *P* values were adjusted for the FDR. ^*^P_FDR_ < 0.05, ^**^P_FDR_ < 0.01, ^***^P_FDR_ < 0.001.

We next assessed proteins associated with the accumulation of AD fibrillar brain pathology over time, that is, Aβ- and tau-PET rate of change. There were no associations between rate of change of Aβ load and any CSF protein level at baseline. On the other hand, higher levels of many core and late upregulated DAPs were related to faster accumulation of tau fibrillar pathology independent of Aβ plaques accumulation (Fig. 4). The proteins mentioned above related to baseline tau-PET load were also the ones most strongly associated with longitudinal tau-PET (FABP3, ENO1, MAPT, NRGN, MIF and GLOD4). Certain proteins such as YWHAQ, DDT, RWDD1, DNPH and TBCA showed stronger associations with the longitudinal tau-PET rate of change than baseline tau-PET load (all standardized coefficients between 0.12 and 0.24, *P*_FDR_ from 0.01 to <0.001; Fig. 4). The 13 proteins most associated with tau-PET rate of change (all *P*_FDR_ ≤ 0.01 in Aβ-positive participants) were all preferentially expressed in neurons, but were not enriched in any biological processes. In the Supplementary Results, we detail analyses based on all Olink proteins.

### The AD proteome is distinct from generalized neurodegeneration

**Fig. 5:**
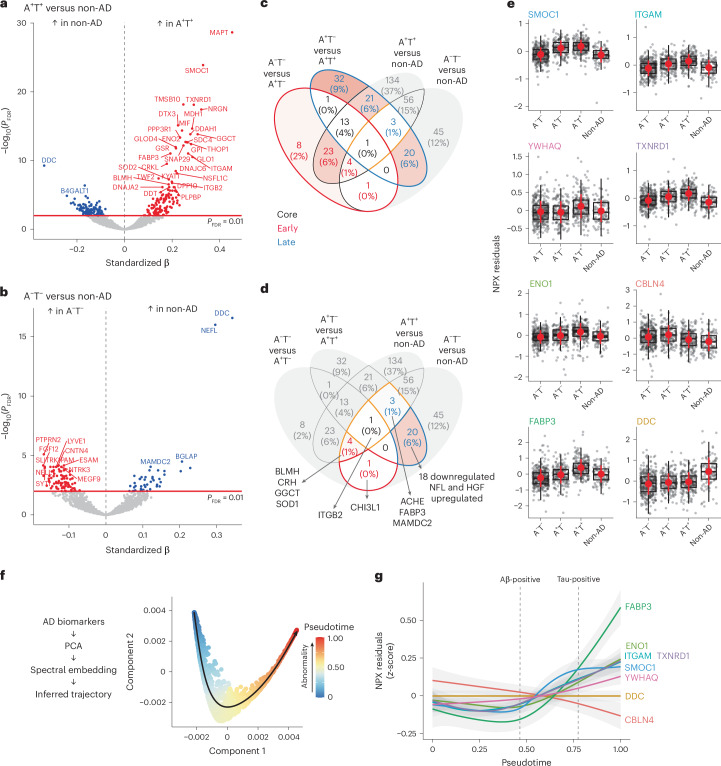
Differentially expressed proteins in non-AD neurodegenerative diseases and trajectories of key proteins. **a**,**b**, Volcano plots depicting DAPs in different groups: A^+^T^+^ (*n* = 231) versus non-AD neurodegenerative diseases (*n* = 110) (**a**) and A^−^T^−^ (*n* = 352) versus non-AD neurodegenerative diseases (**b**). Models included age, sex and mean overall protein level as covariates. The red line represents the threshold of *P*_FDR_ < 0.01, above which we considered proteins for subsequent analyses. **c,d**, Venn diagram summarizing the comparisons shown in **a** and **b** along with those based on the A/T categories in Fig. 2a,b based on proteins significant at *P*_FDR_ < 0.01. The red text corresponds to ‘early’ proteins, the black to ‘core’ proteins and the blue to ‘late’ proteins as per categories defined in previous analyses. **c**, The DAPs highlighted are those related to AD. **d**, The DAPs highlighted are those at the intersections between AD and non-AD neurodegenerative disease. All standardized β values displayed come from two-sided linear regressions and all *P* values were adjusted for the FDR. **e**, Box plots of exemplary DAPs between the different A/T categories as well as non-AD neurodegenerative diseases. The values correspond to residual values after regressing out age, sex and mean overall protein level. In all box plots, the box limits represent the first and third quartiles and the whisker extends to 1.5× the interquartile range. The red dot represents the mean and the red line extends ±1 s.d. **f**, Depiction of steps and spectral embedding from which the inferred trajectory of AD pathology (pseudotime) was estimated. **g**, All proteins shown in **f** plotted against the pseudotime using GAMs. The first dashed line corresponds to Aβ positivity and the second to tau positivity. Error bands around the data correspond to the 95% confidence interval (CI).

**Fig. 6:**
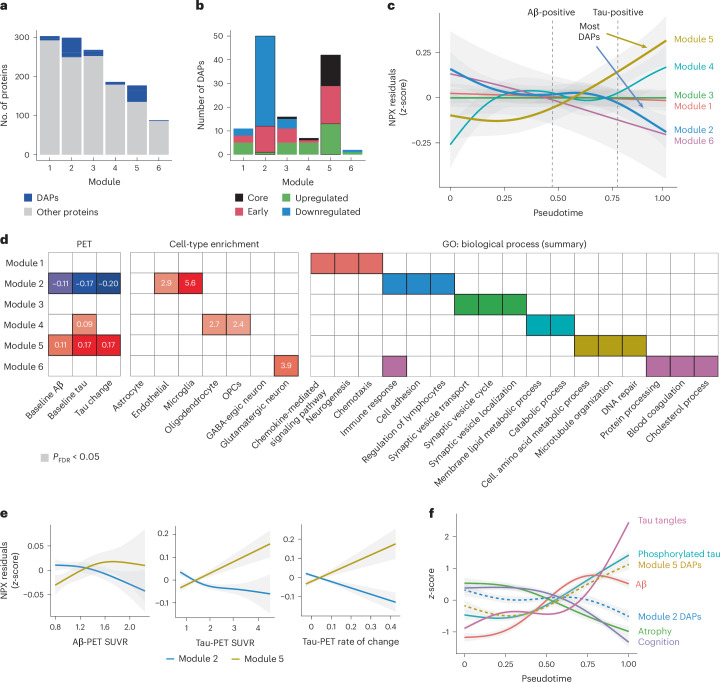
Biological modules derived from protein co-expression. **a**, Bar plots showing the number of proteins in each module derived based on protein co-expression. **b**, Bar plots showing the distribution of the DAPs in each protein co-expression module. **c**, Average protein level from each module plotted against the pseudotime using GAMs. **d**, Summary figure of associations with baseline Aβ- and tau-PET load as well as tau-PET rate of change, cell-type enrichment and summary terms from functional analyses of biological processes for each module. For simplicity, only significant results from two-sided regressions (*P*_FDR_ < 0.05; *P* values were adjusted for the FDR) are displayed in colored cells. **e**, Average protein level from modules 2 and 5 (the two modules containing most DAPs) plotted against Aβ- and tau-PET load (baseline) and tau-PET rate of change (longitudinal) using GAMs. **f**, Key AD markers along with the average DAPs from modules 2 and 5 plotted against the pseudotime using GAMs. The tau tangles were measured from tau-PET SUVR in a temporal meta-ROI, Aβ and phosphorylated tau were measured, respectively, from Elecsys CSF Aβ40/Aβ42 and p-tau181, atrophy was measured from cortical thickness in the temporal lobe and cognition was measured from the MMSE total score. In all panels, error bands around the data correspond to the 95% CI.

Having identified and characterized proteins altered along the A/T continuum, we next sought to establish the specificity of these proteins to AD. To do this, we examined differential protein abundance in the non-AD group (Extended Data Table 1 for all diagnoses). We compared the non-AD group with the A^+^T^+^ group (Fig. 5a), as well as the A^−^T^−^ group (Fig. 5b, see statistical results in Supplementary Table 1), to assess which DAPs in AD were also differentially abundant in other neurodegenerative contexts. The proteins showing the strongest elevation in A^+^T^+^ versus non-AD were all proteins previously identified in analyses restricted to the AD continuum, suggesting their strong specificity to AD. This finding is underscored in Fig. 6c (four-wave Venn diagram), in which 14 of the 15 core proteins were differentially abundant only in the AD continuum (that is, upregulated in A^+^T^−^ versus A^−^T^−^ and in A^+^T^+^ versus both A^+^T^−^ and non-AD). Similarly, almost all early proteins were specific to AD. However, zooming in on proteins that differed in non-AD compared with A^−^T^−^ and in AD (Fig. 5d), we identified DAPs more generally associated with neurodegeneration. A particular intersection of 20 proteins stood out, composed of 18 late downregulated proteins (BSG, CBLN4, CD99L2, CDH6, CHL1, CNTN3, EPHA10, ESAM, GFRA2, LYVE1, MANSC1, MEGF9, NELL1, PAM, PTPRN2, PTPRS, TNFRSF4 and WFDC2) and 2 upregulated late proteins (NfL and HGF). These proteins were differentially abundant in non-AD compared with A^−^T^−^, as well as in A^+^T^+^ compared with A^+^T^−^. Enrichment analyses on the set of significantly less abundant proteins common in the non-AD and the A^+^T^+^ groups highlighted biological processes related to axonogenesis, axon development and neuronal morphogenesis (Supplementary Table 7), supporting the notion that these processes are probably impaired in the later stage of neurodegeneration.

We represented some of the key DAPs pertaining to the different group comparisons in Fig. 5e, with more description of these proteins in Supplementary Results. Of note, DOPA decarboxylase (DDC) was the protein showing the greatest elevation specifically in non-AD compared with both A^−^T^−^ and A^+^T^+^. Higher levels of DDC in the non-AD group was particularly attributable to the high proportion of patients with a Parkinsonian disorder (Supplementary Fig. 3), in line with recent work^21,22^. Some well-established AD-related proteins were also found to be differentially abundant, specifically along the A/T continuum (MAPT and NRGN; core proteins), or were elevated in both the AD and the non-AD groups (NfL and CHI3L1 (YKL-40)), and are displayed in Extended Data 7a.

### Protein trajectories along a pseudotime of AD progression

**Extended Data Fig. 7:**
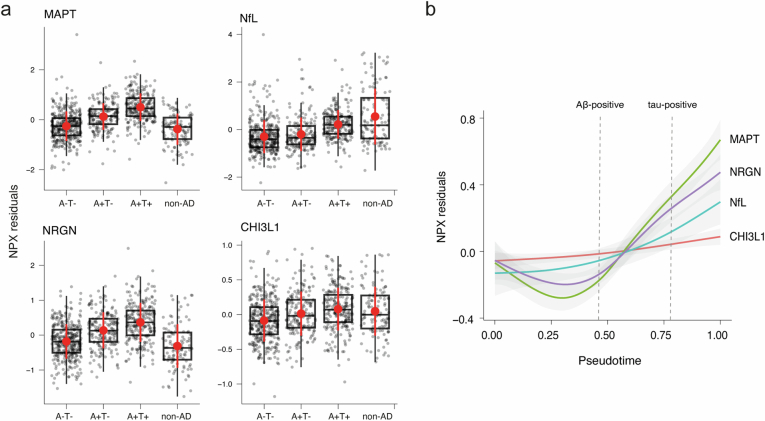
Trajectories of established AD-related proteins. **a**, Box plots of four key AD-related proteins part of the differentially expressed proteins between the different AT categories (A-T-: n = 352; A + T−: n = 184; A + T + : n = 231) and non-AD neurodegenerative diseases (n = 110). Values correspond to residual values after regressing out age, sex and mean overall protein level. In all box plots, the box limits represent the first and third quartile, and the whisker extends to 1.5 time the interquartile range. The red dot represents the mean and the red line extends ±1 standard deviation. **b**, Proteins shown in panel **a** plotted against the pseudotime using generalized additive models. Error bands around the data correspond to the 95% confidence interval.

To provide a granular and sequential measure of advancing AD pathology, we used trajectory inference methods to compare key DAPs on a common continuous scale, representing increasing Aβ and fibrillar tau pathology (Fig. 5f and Supplementary Fig. 4). Each participant was assigned to a point along this AD progression pseudotime. The key individual proteins were then plotted along the pseudotime, revealing how their levels changed along the AD progression (Fig. 5g). SMOC1 (early protein) increased early on and plateaued later in the progression, accurately recapitulating the earlier results from contrasting the different A/T categories. Linear increases in TNXRD1 (core protein) and YWHAQ (late protein) were detected past the point of Aβ positivity. FABP3 also showed rapid elevation, particularly in the last quarter of the pseudotime, with levels exceeding all other proteins. The established AD-related proteins MAPT, NRGN and NfL also showed linear increases past the point of Aβ positivity, with levels being highest for MAPT (Extended Data Fig. 7b).

### Metabolic and inflammatory alterations along the AD spectrum

Protein levels often vary as part of larger networks of co-expressed proteins encompassing different molecular processes. We therefore sought to characterize whether the DAPs that we identified were mostly independent or whether they were particularly salient nodes of larger modules of proteomic changes in response to AD pathology. To do so, we grouped all Olink proteins into biological modules based on protein co-expression using a community detection algorithm^23^. Six principal modules were identified (Fig. 6a and see Supplementary Table 1 for protein assignment to each module). Modules 2 and 5 were particularly enriched with key AD DAPs (50 DAPs in module 2 and 42 in module 5). Module 2 included most of the late downregulated proteins in AD (36 of 48). Module 5 included a mix of core proteins (13 of 15), early and late upregulated DAPs, in similar proportions (Fig. 6b). Plotting each module’s average protein levels along the pseudotime recapitulated the strong association between modules 2 and 5 with the inferred AD trajectory, in opposite directions (Fig. 6c). While levels in module 2 decreased with increasing AD pathology—in line with the high proportion of downregulated proteins composing this module—levels in module 5 increased. Modules 2 and 5 were also most strongly associated with both with baseline and longitudinal Aβ and tau-PET (Fig. 6d,e).

**Extended Data Fig. 8:**
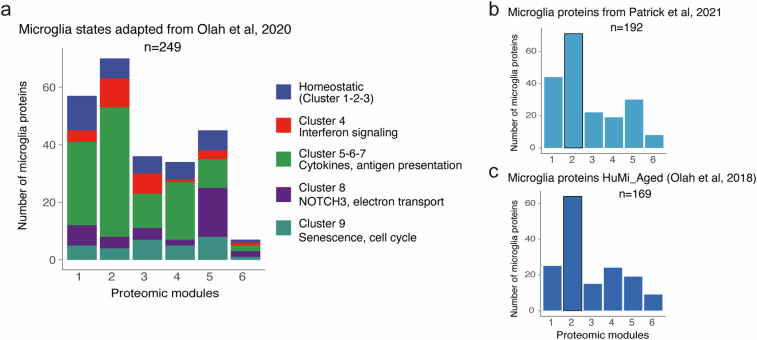
Microglia states across the different protein modules. **a**, Correspondence of different microglial states from human single-cell data from Olah et al, *Nature Communications*, 2020 (stacked barplots) across the different BioFINDER-2 Proteomic modules (x-axis). For simplicity, all homeostatic states have been grouped together, and clusters 5, 6 and 7 have also been grouped together due to their high similarity as described in Olah et al. **b, c**, The predominance of microglia-related proteins in Module 2 was also confirmed in comparing two other sets of microglia genes derived from human aged brains: Patrick et al, *Translational Psychiatry*, 2021 (**b**) and Olah et al, *Nature Communications*, 2018 (**c**). Number of microglial proteins identified from two previous publications (y-axis) across the different Olink modules (x-axis). The number of proteins overlapping between Olink data and each of the three microglia data is reported in the titles.

To better characterize the different modules, cell-type and functional enrichment analyses were also performed (summary in Fig. 6d; all significant terms in Supplementary Table 8). Module 2 was enriched with proteins expressed in microglia and endothelial cells (Extended Data Fig. 5b) and mainly related to immune response, cell adhesion and lymphocyte and leukocyte regulation. A high concentration of inflammatory (cytokines and interleukins) and antigen-presenting microglia proteins were found in this module, based on microglia states previously identified from brain tissue in AD^24^ (Extended Data Fig. 8). Module 5 contained proteins related to amino acid metabolic process, microtubule organization and DNA repair. Module 4 contained only a few DAPs, but was associated with tau-PET and enriched with proteins related to oligodendrocytes and OPCs (Fig. 6c,d). Lastly, half of our module assignment showed consistency with previous CSF proteomic modules, particularly those related to lysosomes, the complement cascade and axonal development (details in Supplementary Fig. 5).

Trajectories of DAPs in modules 2 and 5 were integrated with key markers of AD, such as CSF Aβ42/Aβ40, CSF p-tau181, tau-PET, neurodegeneration (measured with cortical thickness) and cognition (measured with the Mini-Mental State Examination (MMSE)) along the AD pseudotime (Fig. 6f). This visualization helps resolve the differing phases of molecular changes in response to the stereotyped accumulation of AD pathology. Proteins related to metabolic processes (module 5) become abundant in CSF before widespread deposition of fibrillar tau and increase in a manner closely tied to abundance of soluble p-tau in the CSF. Meanwhile, downregulated proteins decrease later in the disease continuum when neurodegeneration and cognitive decline are also present.

## Discussion

In the present study, we identified, validated and characterized several proteins differentially abundant in the CSF of individuals spanning the full spectrum of the AD continuum. We measured the presence and magnitude of AD pathology using Aβ- and tau-PET biomarkers (which quantify fibrillar pathology), both at baseline and over time. Previous proteomic studies had used soluble p-tau as a measure of tau pathology^12,25,26^, but rather it is the tau aggregate pathology that is independently related to neurodegeneration and cognitive decline^15^, highlighting the importance of using tau-PET. Many of the DAPs that we identified were validated in separate datasets and we tested their specificity to AD by additionally contrasting them in patients with other neurodegenerative diseases. We identified distinct sets of proteins that are altered at different stages of the disease, that is, dysregulated early on when participants have only isolated Aβ-plaque pathology versus further altered with the development of fibrillar tau pathology. Many proteins participated in two co-expression networks that became increasingly disturbed alongside the pathological progression of AD and which probably represent key metabolism- and inflammation-related pathways disrupted in AD. Our analyses suggest that the sequential aggregation of Aβ and tau pathologies is mirrored by specific phases of molecular changes and that many of these processes are highly specific to the AD pathophysiological cascade.

The earliest stage of AD involves a continuous accumulation of Aβ-plaque load in the cerebral cortex but without neocortical aggregation of tau tangles^27^. This is a critical stage to study for potential intervention given that cognition and brain integrity remain relatively undisturbed. A specific subset of proteins was already altered in the CSF of individuals within this early AD population. A notable finding from this group included the increased levels of SMOC1 in the CSF, which tracked Aβ-plaque load in the brain, even among participants exceeding the Aβ-positivity threshold. SMOC1 has also been found to be the main protein for which changes in abundance precede Aβ abnormality in autosomal dominant AD^10^, as well as one of the most positively correlated proteins with Aβ-PET in a subset of the ADNI cohort^11^. SMOC1 is expressed in OPCs, is a core component of a biological module related to the extracellular matrix (the ‘matrisome’)^7^ and has been repeatedly found as a key DAP in CSF and brain tissue of patients with AD^8,12,26,28,29^. Recent work demonstrated cell senescence of OPCs in the Aβ-plaque environment, with OPCs adopting an inflammatory phenotype and being unable to differentiate into oligodendrocytes^30^, which might provide insights into the role of SMOC1 in AD. MAPT was also among the proteins that emerged as being already elevated in A^+^T^−^ versus A^−^T^−^, foreshadowing the fibrillar tau deposition that ensued in A^+^T^+^, in line with the different soluble tau variants that reach abnormal levels in CSF or plasma as Aβ plaques become present in the brain^31,32^.

A set of 15 core proteins emerged, the abundance of which in the CSF increased throughout the disease continuum, being elevated in people with isolated Aβ pathology but even further elevated with emergence of cortical fibrillar tau pathology. These proteins included established markers of AD like MAPT (encoding the tau protein) and neurogranin, but also proteins related to immune response (ITGAM, ITGB2 (both expressed in microglia) and MIF), apoptosis (CRKL and MP2K1) and calcium-dependent signaling (PPP3R1, part of the calcineurin complex). These core proteins showed a very high overlap with the proteins changed in autosomal dominant AD^9^; from the top 20 DAPs previously identified in autosomal dominant AD, all except 1 were among the DAPs identified in the present study, with 11 part of the core proteins. These results suggest great commonality between sporadic and autosomal dominant AD and, together with results from other recent proteomic studies^6,12^, support these proteins for improved molecular staging of AD and identification of new therapeutic targets. Furthermore, we found that almost all of these core proteins were specific to AD, because they were also upregulated in the A^+^T^+^ group compared with the non-AD group. The predominance of microglia proteins was also found in one of the co-expression modules changing with AD pathology, highlighting a central component of the immune response in AD.

Proteins altered in the early stages of AD (A^+^T^−^) were enriched in different biological processes, including protein ubiquitination, cellular detoxification, synaptic transmission and response to superoxide species. On inspection of the different proteins individually, the link to oxidative stress was evident for many proteins (DDAH1, GGCT, GSR, PARK7, SOD1 and SOD2), highlighting probable early neural responses to accumulating AD pathology. When contrasting participants with isolated Aβ plaque pathology (A^+^T^−^) to individuals with both cortical Aβ plaque and fibrillar tau pathologies (A^+^T^+^), we found the latter group to be characterized by both increased and decreased abundance of many proteins in the CSF. Several upregulated proteins related to ATP metabolism were independently associated with tau fibrillar load and accumulation (for example, ENO1, ENO2, YWHAQ (member of the 14-3-3 family) and FABP3). As such, dysfunction in energy metabolism is a key alteration in AD pathophysiology and occurs later in the disease progression. The metabolic and glycolytic changes were also reflected in the protein co-expression modules, where the average protein level in such a module was increased late in the disease continuum, when widespread fibrillar pathology is present. The 14-3-3 proteins are also increasingly reported as differentially abundant in AD across studies^11,12^.

Downregulated proteins had seldom been reported in previous studies, where the main comparisons were usually limited to contrasting controls and patients with AD and often without reliable biomarker evidence. The downregulated proteins found only in the A^+^T^+^ group are in line with evidence from cell biology, suggesting that the presence of both Aβ and tau pathologies leads to neuronal hypoexcitability, and thus potentially reduced protein secretion, in opposition to the presence of Aβ alone, which leads to hyperexcitability^33–35^. Also, about half of these proteins were decreased across neurodegenerative diseases, not limited to AD, perhaps indicating involvement in the neurodegenerative process (or response to it) more generally. Several proteins from this group (NELL1, CDH6, CBLN4 and TNFRSF4) were among those for which regional gene expression correlated with the regional tau-PET pattern, but the levels of these proteins in the CSF were not associated with the tau-PET load. We speculate that these proteins may be upstream of tau fibril accumulation, but also acknowledge that protein abundance and RNA expression are not highly correlated^7,36^. As expression of transcripts encoding these proteins was particularly high in regions of the temporal lobe, it might also highlight the vulnerability of these brain regions to neurodegeneration more generally, beyond AD.

The regional distribution of transcripts for the receptor tyrosine kinase MET was also associated with the distribution of fibrillar tau pathology. It is interesting that levels of both MET and its activating ligand HGF (hepatocyte growth factor) were altered in the CSF among individuals with AD pathology. MET and HGF are part of the HGF receptor signaling pathway, a well-established pathway related to apoptosis and cell survival in neurodegenerative diseases^37^. In total, across all DAPs identified, five (HGF, MET, CRLK, MAP2K1 and PLAU) are part of the HGF signaling pathway^38^. These results suggest an important pathway in regulating cell death in AD, and potentially in other neurodegenerative diseases, because MET and HGF were also differentially abundant in non-AD neurodegenerative diseases compared with the A^−^T^−^ group. Further supporting this notion, two ongoing phase 2 trials in patients with mild-to-moderate AD dementia (NCT04488419) and Parkinson’s disease (PD) dementia or dementia with Lewy bodies (NCT04831281) are testing a drug targeting HGF and MET.

Our study has several strengths, including the large sample size and the deeply phenotyped participants with cross-sectional and longitudinal Aβ- and tau-PET, validation in external datasets and comparisons with other neurodegenerative diseases This work was limited to CSF and we acknowledge that future studies integrating CSF and plasma proteomics could help identify more accessible markers of interest to refine disease staging. Patients with AD and non-AD neurodegenerative diseases probably harbor several pathologies and further understanding of the molecular changes underlying different pathologies will be of great interest. Inference drawn from imaging transcriptomics was limited to the few brains available and to molecular changes that happen post mortem. Still, we applied thorough statistical analyses to ensure robustness of the results. Our sample was limited in terms of ethnic and racial diversity, with most participants being self-reported white. In light of recent work highlighting different protein changes with race^39^, it will be important to expand proteomic studies to more diverse populations.

Overall, using a multi-omics approach, we provided a new insight on key proteins and molecular pathways that co-occur with, and follow the accumulation of, Aβ and tau load along the AD continuum. Our study highlights the importance of focusing on the underlying pathology, because certain groups of proteins could be uncovered specifically in relation to isolated Aβ-plaque pathology, to both Aβ-plaque and tau tangle pathologies, or were increasingly altered as pathology increased. A portion of these proteins was also altered in other neurodegenerative diseases, suggesting in part common pathways at play leading to degeneration. The comprehensive analyses yielded new potential targets for therapeutic approaches at different stages of the disease.

## Methods

### Participants: BioFINDER-2 cohort

Participants were part of the ongoing prospective Swedish BioFINDER-2 cohort (NCT03174938, http://www.biofinder.se), which spans the full spectrum of the AD continuum, ranging from adults with intact cognition or subjective cognitive decline, MCI to AD dementia, as well as patients with non-AD neurodegenerative diseases. All participants were recruited at Skåne University Hospital and the Hospital of Ängelholm, Sweden. The main inclusion criteria were, as described previously^40^, age ≥40 years, fluency in the Swedish language, MMSE between 27 and 30 or 26 and 30 depending on age of CU participants, between 24 and 30 for MCI and ≥12 for patients with AD dementia. Exclusion criteria include unstable systemic illness, neurological or psychiatric illness, alcohol or substance abuse or refusing a lumbar puncture or neuroimaging. MCI diagnosis was established if participants performed <1.5 s.d. below the normative score on at least one cognitive domain from an extensive neuropsychological battery examining verbal fluency, episodic memory, visuospatial ability and attention/executive domains^41^. AD dementia diagnosis was determined using the criteria for dementia caused by AD from the *Diagnostic and Statistical Manual of Mental Disorders*, 5th edn (DSM-5)^42^ and, if a positive diagnosis was made, this was confirmed using Aβ biomarkers based on the updated National Institute on Aging and Alzheimer’s Association (NIA-AA) criteria for AD^43^. The group of non-AD neurodegenerative diseases was composed of patients who fulfilled criteria for dementia owing to frontotemporal dementia, PD with dementia, subcortical vascular dementia, PD, progressive supranuclear palsy, multiple system atrophy, corticobasal syndrome or primary progressive aphasia. Clinical diagnosis was determined according to the main criteria for each disease, as detailed elsewhere^40^, and was done either at baseline or during the course of follow-up visits. The study was approved by the Regional Ethics Committee in Lund, Sweden. All participants gave written informed consent to participate and they were compensated for each study visit. All data for the current study were acquired between April 2017 and December 2022. All participants included in the present study had complete proteomic data and CSF Aβ42/Aβ40 and tau-PET measures available at baseline, totaling 935 individuals.

### CSF proteomic measures

CSF samples from baseline visits were analyzed with a validated, highly sensitive and specific Olink (Uppsala, Sweden) proteomic assay, an antibody-based Proximity Extension Assay^44^. In BioFINDER-2, a total of 2,943 proteins, corresponding to Olink Explore 3072, were measured across 8 multiplex protein panels (oncology, neurology, cardiometabolic, inflammation, oncology II, neurology II, cardiometabolic II, inflammation II), each containing 367–369 proteins. All measurements were performed by the company, blinded to any information on the samples. Samples were randomized across plates and appropriate controls were included on each plate, to allow for thorough quality control (QC) as per the Olink technology (see ref. ^45^ for all details). Samples that did not meet thorough QC received a warning label. Each assay had a lower limit of detection (LOD) provided, defined as 3 s.d. above background (based on three negative controls included on every plate). Protein levels were reported as normalized protein expression (NPX) values, a relative quantification unit corresponding to a log_2_ scale provided by Olink. Only proteins for which at least 70% of participants had levels above LOD were retained for further analysis, resulting in 1,331 proteins. Distribution of the proteins meeting the LOD criteria across the different Olink panels is provided in Supplementary Table 9. From these proteins, a few datapoints had an assay warning label (about 0.2% of the data, 46–64 measurements on 37 proteins) and were excluded from analyses, but all datapoints below the LOD were kept in analysis, as per the Olink recommendations and in line with recent analyses^6,46^. Proteomic data distribution was assumed to be normal but this was not formally tested. We noted a clear interaction between *APOE*4 genotype and apoprotein E (ApoE) levels in Olink and provided all analyses to that effect in Supplementary Results and Supplementary Figs. 1 and 2. Given that we cannot yet explain this effect, we have removed ApoE from the main analyses and discuss it in the Supplementary Results section ‘Olink CSF ApoE results’. We should note that results from all proteins except ApoE were not influenced by *APOE*4 genotype.

### Mean overall protein level as a covariate

We also calculated a measure of the mean overall protein levels, to be included in all analyses as a nuisance covariate. The rationale to include such a measure was to account for interindividual variability in CSF dilution levels, which could be the result of different rates of clearance or production. We recently showed that nearly half of the individual protein levels correlated highly with the overall protein average^47^, making it an important confounder to consider in proteomic analyses. For instance, for a given individual, higher or lower values across proteins might depend on their overall CSF level and, as such, accounting for this CSF dynamics should be considered. In the present study, we calculated the average *z*-scored NPX values from all highly detected proteins, that is, proteins for which levels for >90% of participants were above LOD (*n* = 1,157), to ensure that this overall average reflected proteins measured with high confidence. We refer to this measure as the mean overall protein level and it was included as a covariate in all analyses, along with age and sex.

### CSF markers of Aβ

CSF Aβ42 and Aβ40 were measured using the Elecsys immunoassays (Roche Diagnostics)^48^. A pre-established cutoff of 0.08 on the CSF Aβ42/Aβ40 ratio was used to define Aβ positivity based on Gaussian mixture modeling^40^. A minority of participants did not have Elecsys measurements available, in which case clinical routine assays and pre-established cutoffs were used (*n* = 31 with the Lumipulse G assay, cutoff of 0.072 (ref. ^49^); *n* = 20 with Meso Scale Discovery, cutoff of 0.077). Participants below the cutoffs were considered to be Aβ positive (A^+^), which was used to form the different A/T groups.

### PET acquisition and processing

Aβ- and tau-PET images, acquired on digital GE Discovery MI scanners, were available in the BioFINDER-2 cohort. For tau-PET, acquisition was done 70–90 min after injection of ~370 MBq of [^18^F]RO948 and was available for all participants. For Aβ-PET, acquisition was done 90–110 min after injection of ~185 MBq of [^18^F]flutemetamol. As per the study protocol, patients with dementia did not undergo Aβ-PET. Images were processed according to our pipeline, described previously^50^. Briefly, PET images were attenuation corrected, motion corrected, summed and registered to the closest T1-weighted magnetic resonance (MR) image processed through the longitudinal pipeline of FreeSurfer v.6.0. Structural T1-weighted images were acquired from a magnetization-prepared rapid gradient echo (MPRAGE) sequence with 1 mm isotropic voxels on a Siemens 3T MAGNETOM Prisma scanner (Siemens Healthineers). The SUVR images were created using the inferior cerebellar gray matter as the reference region for [^18^F]RO948 and the whole cerebellum for [^18^F]flutemetamol^16^.

Both continuous values and binary classification from key regions were used in different analyses. For Aβ-PET, the average SUVR was calculated from a global neocortical ROI, including prefrontal, lateral temporal, parietal, anterior cingulate and posterior cingulate/precuneus, and the cutoff for positivity was SUVR = 1.03, defined from Gaussian mixture modeling^16^. For tau-PET, the average SUVR was calculated in a temporal ROI composed of the entorhinal cortex, amygdala, parahippocampal gyrus and inferior temporal and middle temporal gyri^51^. A cutoff of 1.36 SUVR was defined from Gaussian mixture modeling, which was the same cutoff if calculating 2 s.d. from the mean of CU Aβ-negative individuals. Participants above the cutoff were considered tau-positive (T^+^), which was used to form the different A/T groups throughout the article.

A subset of participants also had longitudinal Aβ- and tau-PET data available. For these participants, we derived individual rate of change by fitting linear mixed-effect models with random slope and intercept using the R package lme4 v.1.1-31, where PET SUVR was the dependent variable and time in years from the baseline scan date was the independent variable. The slope of each participant from those models was used to represent Aβ- or tau-PET SUVR change per year. Participants had between two and four follow-up scans. We focused subsequent analyses on participants who had both Aβ and tau rate of change, which resulted in 458 CU and MCI participants with, on average, 2.6 ± 1.0 years of follow-up (range: 1.3–4.4 years).

### Differential protein expression analyses

Linear models were used to establish DAPs between A/T groups, including age, sex and the mean overall protein level as covariates. The first set of comparisons captured the AD continuum: an A^−^T^−^ versus A^+^T^−^ contrast captured DAPs in the early stages of AD progression, whereas an A^+^T^−^ versus A^+^T^+^ contrast captured DAPs associated with later stages of biomarker progression. Next, separate models contrasting Aβ-negative patients with non-AD neurodegenerative diseases to A^−^T^−^ and A^+^T^+^ were performed, to investigate proteins specific to AD as well as proteins generally related to neurodegeneration. The A^−^T^+^ category included only six participants (three CU, three MCI), and thus this category was not included in analyses. Some 52 participants with non-AD neurodegenerative diseases were Aβ positive and were also excluded, to capture changes independently as much as possible from AD pathology in the non-AD group. Ultimately, differential expression analysis was performed on 877 participants and the results are presented as volcano plots. Analyses were adjusted for multiple comparisons using the Benjamini–Hochberg method (FDR). Given that one of our main objectives was to characterize the strongest DAPs, we focused subsequent analyses on DAPs that were significant at *P*_FDR_ < 0.01.

### Validation of key DAPs in independent datasets

To further validate some of the key proteins identified in the main cohort (BioFINDER-2), we analyzed a subset of BioFINDER-1 participants (CU older adults and patients with MCI) who also had Olink proteomic data from CSF available, although fewer proteins and different panels were available (neuro-exploratory, neurology, inflammation and cardiovascular III). BioFINDER-1 (NCT01208675) is an older prospective cohort in which participants were enrolled between 2010 and 2014, with similar inclusion and exclusion criteria as for BioFINDER-2 (ref. ^52^). Participants (*n* = 631) who had proteomic data and CSF Aβ42/Aβ40 to determine their Aβ status were included. We analyzed the proteins overlapping with those available in the main cohort, for a total of 202 proteins. Tau-PET was not available in this cohort. We thus performed differential expression analyses comparing A^−^ with A^+^ participants.

We also validated some of the key proteins in ADNI, where CSF proteomic levels of approximately 7,000 proteins were measured with SomaScan 7K (v.4.1) for 758 individuals across all ADNI studies, as described previously^12^. We selected participants who had proteomic data available within an 18-month window from Aβ-PET, yielding 463 participants for analyses. ADNI is a multisite study launched in 2003 as a public–private partnership. The primary goal of ADNI has been to test whether serial MRI, PET, other biological markers and clinical and neuropsychological assessment can be combined to measure the progression of MCI and early AD. For up-to-date information, see www.adni-info.org. Analyses were done on proteins that overlapped with those available in BioFINDER-2, for a total of 1,438 aptamers. Positivity for Aβ-PET was taken from the ‘AMYLOID_STATUS’ variable, reflecting different tracer-dependent thresholds for florbetapir or florbetaben, based on the most updated methods provided by ADNI (see Amyloid PET processing methods v.2, revised 29 June 2023). Tau-PET was not available in the participants with proteomics. We thus performed differential protein abundance analyses between A^−^ and A^+^ participants including age and sex as covariates.

### Imaging transcriptomics relating regional gene expression and AD pathology

To further investigate the relationship between key DAPs and AD pathology, we performed region-wise association between gene expression of the DAPs across brain regions and average Aβ- and tau-PET across the same brain regions. Gene expression was generated using the regional microarray expression data obtained from six postmortem brains provided by the AHBA^53^ (https://human.brain-map.org). Data were processed with the abagen toolbox v.0.1.3 (ref. ^54^) (https://abagen.readthedocs.io/en/stable/index.html), a Python-based package to access and work with the Allen Brain data microarray expression data. Briefly, processing steps involved collapsing data into ROIs (parcels of a specified brain atlas) and combining across donors. Regional microarray expression data were generated for two brain atlases—the Desikan–Killiany atlas and the Schaefer 200 parcels—both in MNI (Montreal Neurological Institute) space. The abagen default parameters were used, with the exception that, for the Desikan–Killiany atlas, gene normalization was done within structural classes (cortical, subcortical/brainstem), given that gene expression can differ in cortical versus subcortical regions^53,55^. All details of the abagen workflow for generating these data are as follows: first, microarray probes were reannotated using data provided by Arnatkevic̆iūtė et al.^55^; probes not matched to a valid Entrez ID were discarded. Next, probes were filtered based on their expression intensity relative to background noise^56^, such that probes with intensity less than the background in ≥50% of samples across donors were discarded, yielding 31,569 probes. When multiple probes indexed the expression of the same gene, we selected and used the probe with the most consistent pattern of regional variation across donors (that is, differential stability). In the present study, regions correspond to the structural designations provided in the ontology from the AHBA. The MNI coordinates of tissue samples were updated to those generated via nonlinear registration using the Advanced Normalization Tools. Samples were assigned to brain regions in the provided atlas if their MNI coordinates were within 2 mm of a given parcel. To reduce the potential for misassignment, sample-to-region matching was constrained by hemisphere and gross structural divisions (that is, cortex, subcortex/brainstem and cerebellum, such that, for example, a sample in the left cortex could be assigned only to an atlas parcel in the left cortex^55^). All tissue samples not assigned to a brain region in the provided atlas were discarded, yielding a total of 83 regions. Intersubject variation was addressed by normalizing tissue sample expression values across genes using a robust sigmoid function and then rescaled to the unit interval. Gene expression values were then normalized across tissue samples using an identical procedure. Normalization was performed separately for samples in distinct structural classes (that is, cortex, subcortex/brainstem and cerebellum). Samples assigned to the same brain region were averaged separately for each donor and then across donors, yielding a regional expression matrix with brain regions as rows and genes as columns.

To get regional levels of AD neuropathology, for each region of the Desikan–Killiany (all cortical regions, hippocampus and amygdala) and Schaefer 200 atlases, we averaged Aβ- and tau-PET across Aβ-positive participants. The main correlational analyses were performed across all DAPs previously identified. To ensure that analyses were not driven by statistical autocorrelations, we performed null modeling with BrainSMASH (https://brainsmash.readthedocs.io/en/latest/index.html), a Python-based package for statistical testing of spatially autocorrelated brain maps^57^. BrainSMASH simulates surrogate brain maps with spatial autocorrelation that matches the spatial autocorrelation of the original brain map of interest, in the present study the Aβ- or tau-PET map. We used a Euclidean distance matrix of each atlas as input to generate 1,000 surrogate maps preserving autocorrelation, otherwise using the default BrainSMASH parameters. Pearson’s correlation was then computed region-wise between (1) the original maps of interest, that is the Aβ/tau-PET and the gene expression maps, and (2) each surrogate PET map and the original gene expression map, to create a null distribution. Nonparametric *P* values were then computed, corresponding to the frequency that correlation with the surrogate maps exceeded the observed correlation with the original gene expression map. Genes were considered to have expression profiles significantly related to pathology when they had BrainSMASH-corrected *P* values < 0.05 across both atlases.

### Cell-type enrichment analysis

To measure cell-type expression of the different genes of interest, we used the RNA sequencing (RNA-seq) data from the middle temporal gyrus (MTG) available from the Allen Brain Atlas consortium (Human MTG 10x SEA-AD 2022, https://portal.brain-map.org/atlases-and-data/rnaseq/human-mtg-10x_sea-ad), applying a similar approach to that described previously^58^. This dataset includes single-nucleus transcriptomes from 166,868 total nuclei of five postmortem human brain specimens. We downloaded the Seurat object and applied the function AverageExpression from the R package Seurat v.4.3.0 (refs. ^59,60^) to generate cell-type expression levels. Using the class and subclass annotation available from the Allen Brain data, we calculated the average expression from non-neuronal cells (microglia, astrocytes, oligodendrocytes and OPCs) and neuronal cells (GABA-ergic neurons and glutamatergic neurons), after removing the annotations labeled ‘None’. We then calculated the percentage expression across all these cell types from the average expression.

We also performed cell-type enrichment analysis using the Allen Brain MTG data as the cell-type dataset in the R package Expression Weighted Cell Type Enrichment (EWCE) v.1.6.0 (ref. ^61^). We performed bootstrap cell-type enrichment analyses (*n* = 1,000), providing a list of key gene hits and defining the background set at the 1,331 Olink proteins used across analyses. The cell types included were microglia, astrocytes, oligodendrocytes, OPCs, GABA-ergic neurons and glutamatergic neurons. Enrichment was considered significant if the surviving adjustment for multiple comparisons was based on a *P*_FDR_ < 0.05 (Benjamini–Hochberg method).

### Differential gene expression

We used the recently published single-nucleus dataset of 2.3 million nuclei from the prefrontal cortex of 427 ROSMAP participants to investigate differential gene expression of some of the key proteins identified in relation to postmortem AD pathology^20^. Differential gene expression across all cell types and AD pathology was downloaded from Github (https://github.com/mathyslab7/ROSMAP_snRNAseq_PFC/tree/main/Results/DecontX_RUVr_Differential_gene_expression_analysis/muscat_DecontX/Results). We considered differential gene expression in relation to pathology to be significant for *P*_FDR_ < 0.05, correcting for multiple comparisons across all cell types and all measures of pathology.

The average gene expression within each of the main cell types (microglia, astrocytes, oligodendrocytes, OPCs, GABA-ergic neurons and glutamatergic neurons) was also generated in this dataset using Seurat as described above. Those measures were derived using the processed data (single-nucleus RNA sequencing (snRNA-seq) 10×) for each cell type available on Synapse (https://www.synapse.org/#!Synapse:syn52293433). We generated the average expression across (1) the whole dataset of 427 participants, (2) only A^−^ donors based on a CERAD (Consortium to Establish a Registry for Alzheimer’s Disease) score of possible or no AD and (3) only A^+^ donors based on a CERAD score of probable or definite.

### Functional enrichment analyses

For functional enrichment analyses, we used the WEB-based Gene SeTAnaLysis Toolkit (WebGestalt: https://www.webgestalt.org)^62,63^. We performed human over-representation analyses using the GO database for biological process (BP) and cellular component (CC), defining the background set as the 1,331 Olink proteins used across analyses. We used the default parameters from WebGestalt and reported only terms that survived adjustment for multiple comparisons based on a *P*_FDR_ < 0.05 (Benjamini–Hochberg method). To reduce term redundancy in displaying the main results, we used the affinity propagation option from the R package apcluster to cluster gene sets. All individual significant GO terms are provided in Supplementary Tables 6–8.

### Inferred trajectory using pseudotime

We created a pseudotime variable based on AD pathology, to be able to compare protein trajectories along a continuous measure of AD progression. The method used was adapted from Tasaki et al.^36^. Input variables for the pseudotime included state-of-the-art biomarkers of AD pathology which showed the greatest availability across the full sample of BioFINDER-2 participants (*n* = 1504), to derive the AD pseudotime as robustly as possible. Specifically, we used (1) plasma tau phosphorylated at Thr217, which has recently been shown to be slightly better related to Aβ pathology in the brain than CSF Aβ42/Aβ40 (ref. ^32^) and (2) tau-PET SUVR from three meta-ROIs representing Braak stages^64^ (Braak I–II, III–IV and V–VI). First, we applied principal component analysis (PCA) to the different AD biomarkers and the four PCs were included as input to a spectral embedding analysis. These steps were implemented in the scikit-learn package^65^ (v.0.24.2) in Python. The spectral embedding analysis reduced the data nonlinearly to a latent two-dimensional (2D) space in which individuals were embedded based on their biomarker values. We inferred a trajectory across this 2D space using SCORPIUS^66^ v.1.0.8, a package on R to infer chronologies in an unsupervised manner, which is one of the best performing tools in trajectory inference methods^67^. Briefly, SCORPIUS partitioned samples into clusters and optimized the shortest and smoothest path going through the center of these clusters. Each datapoint (participant) is thus assigned a coordinate along this pseudotime trajectory, going from 0, representing the lowest levels of pathology, to 1, representing the highest levels of pathology. Individual key proteins or the average of protein modules was then plotted against the pseudotime using smoothed generalized additive model (GAM) lines from ggplot2 to estimate the trajectories of DAPs across a granular estimate of AD progression.

### Co-expression modules analysis

We derived modules of co-expressed proteins using the consensus clustering algorithm SpeakEasy, which allows for robust community detection. We regressed out age, sex and mean overall protein level from each protein level (the covariates used across all analyses) and used the standardized residuals from each protein to generate a protein–protein correlation matrix based on Spearman’s correlation (1,331 × 1,331 proteins), which served as input for clustering. We also applied PCA to Spearman’s correlation matrix of the residual protein data to investigate the data organization. The first PC, explaining 13% of the variance, showed a strong anti-correlation with protein–protein correlation, potentially masking more modular and disease-relevant signal. We thus further regressed PC1 from the data before applying SpeakEasy. The investigator was blinded to any sample and protein label when deriving the modules. We computed whole-module expression by taking the mean protein level across all proteins within each module for each participant.

### Immunofluorescent staining from postmortem brain tissue

Immunofluorescent staining from entorhinal cortex tissue from six patients with clinically diagnosed AD (*n* = 6, age 68–91 years, three women, Braak IV–VI, all with elevated amyloid plaques) and six nondemented controls (*n* = 6, age 72–92 years, five women, all Braak I or II, no or some amyloid plaques) from the Netherlands Brain Bank was performed for SMOC1, as well as markers for AD pathology (plaques and neurofibrillary tangles).

All donors provided informed consent for brain autopsies and the use of material and clinical data for research purposes, in compliance with national ethical guidelines. The samples were immersion-fixed in 4% paraformaldehyde on autopsy and left in phosphate-buffered saline (PBS) with 30% sucrose for 3 d. Thereafter the tissue was sectioned with a microtome (Leica SM 2010R) into 40-µm-thick sections and kept free floating in antifreeze solution at −20 °C.

For immunofluorescent staining against SMOC1 (Merck, cat. no. WH0064093M3, clone 8F10), p-tau 231 (Abcam, cat. no. ab151559) and methoxy-X04 (Tocris Biotechne, cat. no. 4920), sections of entorhinal cortex were incubated in citrate buffer 30% at 80 °C. After rinsing in potassium PBS (KPBS) the sections were incubated in blocking solution (5% normal goat serum in KPBS) at room temperature followed by an incubation in primary antibodies in blocking solution overnight at 4 °C. Afterwards, the tissue was washed with KPBS, 0.25% Triton X-100 and incubated in the appropriate secondary antibody (goat anti-rabbit 488, 1:200 (Invitrogen, cat. no. A11008) and goat anti-mouse 549, 1:200 (Invitrogen, cat. no. A11029)) in blocking solution for 24 h at 4 °C. After washing with KPBS, the tissue was stained with the ligand methoxy-X04 diluted 1:5,000 in PBS for 1 h at room temperature.

We compared optical density (OD) area fraction between AD and nondemented controls. Analysis of the SMOC1-immunostained area fraction was performed by capturing six images of the entorhinal cortex in each donor with an Olympus BX41 light microscope with ×40 objective (72 fields in total). The area fraction of SMOC1 in each image was analyzed using imageJ v.1.54g and the values of each case were averaged and presented as percentage OD area fraction.

### Statistics and reproducibility

All analyses were performed in R v.4.2.1 or Python v.3.9.2. All plots were generated with the R package ggplot2 v.3.4.0. Brain renderings were generated with the Connectome Workbench software v.1.5.0. All regression analyses included age, sex and mean overall protein level as covariates. No statistical method was used to predetermine sample size; we used all participants who had proteomic and biomarker data available. Plots displaying levels of individual proteins or average from modules correspond to residual NPX values when regressing out the three covariates (age, sex and mean overall protein level). All statistical tests were two sided and *P* values adjusted for FDR.

### Reporting summary

Further information on research design is available in the Nature Portfolio Reporting Summary linked to this article.

## Online content

Any methods, additional references, Nature Portfolio reporting summaries, source data, extended data, supplementary information, acknowledgements, peer review information; details of author contributions and competing interests; and statements of data and code availability are available at 10.1038/s41593-024-01737-w.

## Supplementary information

Supplementary Information Supplementary Methods, Tables 1–8, Results and Figs. 1–5.

Reporting Summary

Supplementary Table 1 Supplementary Table 1 Summary statistics of differential protein abundance across the different A/T categories. Supplementary Table 2 Summary statistics of differential protein abundance between A^−^ and A^+^ participants in BioFINDER-1 (validation cohort). Supplementary Table 3 Summary statistics of differential protein abundance between A^−^ and A^+^ participants in ADNI (validation cohort). Supplementary Table 4 Summary statistics of imaging transcriptomics using BrainSmash. Supplementary Table 5 Summary statistics of associations between DAPs and Aβ- and tau-PET. Supplementary Table 6 GO significant terms from enrichment analyses of early, core and late proteins. Supplementary Table 7 GO significant terms from enrichment analyses of the downregulated proteins in A^+^T^+^ and non-AD. Supplementary Table 8 GO significant terms from enrichment analyses in the co-expression modules.

## Figures and Tables

**Extended Data Table 1:**
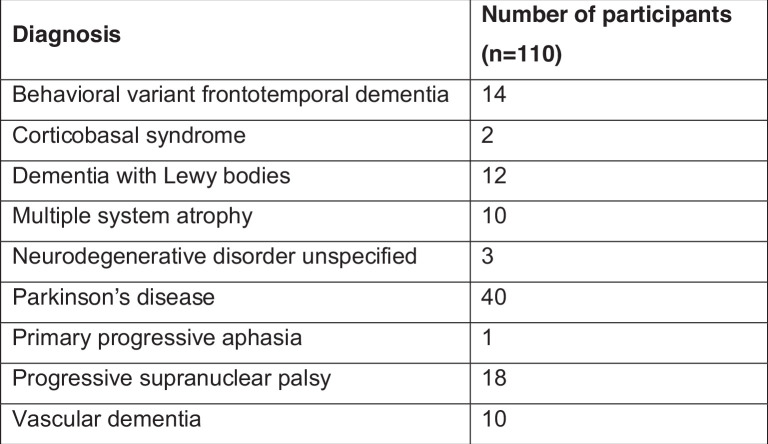
Clinical diagnoses of the non-AD group individuals

**Extended Data Table 2:**
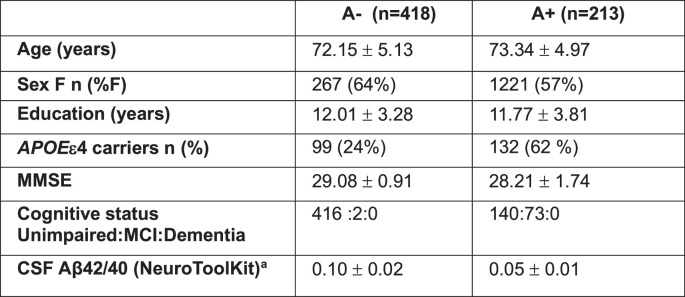
Validation cohort (BioFINDER-1) demographics

Data are presented as mean ± s.d. unless specified otherwise. **a**, Aβ status (positive or negative) based on CSF Aβ42:Aβ40 ratio,

^a^The 12 and 10 missing values in the two categories for which clinical assays were used to determine the Aβ status.

## Data Availability

Pseudonymized data from BioFINDER (Principal Investigator: O.H.) can be shared with qualified academic researchers after a request for the purpose of replicating procedures and results presented in the present study. In line with the EU General Data Protection Regulation legislation, a data transfer agreement must be established with Skåne University Hospital (Region Skåne) to share data. The agreement will include terms of how data are stored, protected and accessed, and define what the receiver can or cannot do. The proposed analyses must be compliant with decisions made by the Swedish Ethical Review Authority. This procedure is in place to ensure the anonymity of the participants, who have not consented to open sharing of their information, and to ensure that all data analyses are restricted to the ones agreed by the study participants and the Swedish Ethical Review Authority. ADNI data used in this manuscript are publicly available from the ADNI database (adni.loni.usc.edu) on registration and compliance with the data use agreement. SnRNA-seq data from ROSMAP are available at https://www.synapse.org/#!Synapse:syn52293433 on data use agreement. SnRNA-seq from the Allen Brain Institute is openly available at https://portal.brain-map.org/atlases-and-data/rnaseq. Summary statistics from all analyses are provided in the Supplementary Information.

## Code availability

All code was written using available packages in R and Python and has been provided at https://github.com/alexapichet/NatureNeuro_2024_proteomics.
