# Supplementary material for: Proteomic changes in Alzheimer’s disease associated with progressive Aβ plaque and tau tangle pathologies
Source: Nat Neurosci. 2024 Aug 26;27(10):1880–91. doi: 10.1038/s41593-024-01737-w (PMC11452344; doi:10.1038/s41593-024-01737-w)
Supplement: Supplementary file 1 — Supplementary Methods, Tables 1–8, Results and Figs. 1–5. [file 41593_2024_1737_MOESM1_ESM.pdf]

nature neuroscience

Supplementary information

https://doi.org/10.1038/s41593-024-01737-w

Proteomic changes in Alzheimer's disease associated with progressive Aβ plaque and tau tangle pathologies

In the format provided by the authors and unedited

Supplementary Material

RESULTS

Supplementary Results - Regional gene expression and PET pattern across DAPs

Supplementary Results - Proteins presage subsequent tau-PET accumulation

Supplementary Results - Selected proteins levels across groups

Supplementary Results - Olink CSF ApoE results

FIGURES

Supplementary Fig. 1. CSF ApoE levels by *APOE* genotype

Supplementary Fig. 2. Differential protein abundance analyses adjusting for *APOE*4 genotype

Supplementary Fig. 3. DDC levels across diagnoses in the non-AD group

Supplementary Fig. 4. Aβ and tau markers mapped on the 2D representation of the pseudotime

Supplementary Fig. 5. Comparison of modules correspondence with previously published WGCNA modules

TABLE

Supplementary Table 9 - Olink panel statistics

SUPPLEMENTARY RESULTS

Regional gene expression and PET pattern across DAPs

We investigated if the regional gene expression pattern of DAPs were related to the pattern of the Aβ and tau aggregates in the brain (Extended Data Fig. 1). To do so, we correlated the regional gene expression in the brain of each DAP with group-level of Aβ- and tau-PET deposition in the same regions. We acknowledge that regional RNA expression does not directly represent secretion of the proteins in the CSF and these analyses were meant to further investigate the DAPs. Among the 128 proteins tested, there were six for which regional protein coding gene expression matched the tau-PET pattern (*NELL1, MET, CBLN4, CPLX2, TNFRSF4, TXNRD1*; all correlations between 0.53 and 0.36 and all $p<0.01$) and one protein, *CDH6*, that inversely matched the tau-PET pattern (correlation of -0.42), consistently across two brain atlases and after accounting for spatial autocorrelation (Extended Data Fig. 1a, see Supplementary Table 4 for all statistical results). The gene expression pattern of *MAPT* (microtubule-associated protein tau) was significantly related to tau-PET deposition in the Desikan Killiany atlas but not in the Schaefer atlas (correlation of 0.36 and 0.19 respectively). With Aβ, only *CRH* (correlation of 0.55) showed correspondence between regional gene expression pattern and Aβ-PET SUVR consistently across the two atlases (Extended Data Fig. 1b). Overall, imaging transcriptomics revealed only few and moderate associations between regional RNA expression of certain DAPs and the tau-PET pattern.

Proteins presage subsequent tau-PET accumulation

In complementary analyses, to not miss any key hits associated with accumulation of tau fibrils, we repeated the same regression models as shown in Fig. 4 across all 1331 Olink proteins. All significant proteins were part of the 128 DAPs previously identified, except one, BPM4 (Bone Morphogenetic Protein 4, a secreted ligand of the TGF-beta family of proteins, mainly expressed in glutamatergic neurons) that was associated with *reduced* tau-PET rate of change in all CU and MCI, as well as in Aβ-positive participants only (standardized coefficients of -0.18 and -0.28, p_{FDR} of 0.01 and 0.05, respectively). Of note, higher levels of TXNRD1 – where the regional gene expression matched the regional average tau-PET uptake (Extended Data Fig 1a) – was related to higher tau-PET rate of change over time and uptake at baseline (standardized coefficient of 0.18 and 0.14 respectively, p_{FDR}=0.02).

Selected proteins levels across groups

We represented some of the key DAPs pertaining to the different group comparisons in Fig. 5e: SMOC1 (expressed in OPCs) was a protein particularly and uniquely elevated early in the AD process (already elevated in A+T-). SMOC1 was not differentially abundant in the other (non-AD) neurodegenerative group, and no further change in protein abundance occurred with development of fibrillar tau pathology later in the AD process (A+T+). ITGAM (expressed in microglia) was one of the core AD proteins increased both in A+T- and further increased A+T+, whereas YWHAQ, TXNRD1 (expressed in glutamatergic neurons) and ENO1 (expressed in astrocytes and glutamatergic neurons) were late upregulated proteins in AD (increased in A+T+ compared to A+T-) that were unchanged in other (non-AD) neurodegenerative diseases. CBLN4 and FABP3 (expressed in glutamatergic neurons) were proteins that changed later in the AD continuum, being respectively decreased and increased in A+T+, but which were also differentially abundant in non-AD compared to A-T-, suggesting a relationship to neurodegeneration more generally. Lastly, DOPA decarboxylase (DDC, expressed in similar proportion in glutamatergic and GABAergic neurons) was the protein showing the greatest elevation specifically in non-AD compared to both A-T- and to A+T+.

CSF Olink ApoE results

Using the Olink Explore 3072 panel in BioFINDER-2, we observed lower levels of ApoE in the A+T- compared to the A-T- participants in CSF. We could not reproduce this effect in ADNI, which used the SomaLogic 7K pattern. In BioFINDER-2, *APOE*4 carriers had lower levels of ApoE, whether measured across the AD continuum or in a restricted sample of younger A- participants (age 20-50) (Supplementary Fig. 1). Further, when repeating differential abundance analyses with *APOE*4 status included as a covariate, all results remained consistent except for ApoE, which was no longer significantly different between A+T- and A-T- groups (Supplementary Fig. 2a-b). Given the imbalance of *APOE*4 carriers between A+T- and A-T- and groups, we also repeated analysis in a subsample matched for *APOE*4 carrier proportion. Again, all results remained consistent except for ApoE (Supplementary Fig. 2c). Since there is a strong and unexplained genotype and platform interaction on ApoE, and given its importance in the AD pathophysiological cascade, we have opted to remove it from the proteins presented in the main text.

Supplementary Figure 1. CSF ApoE levels by *APO*E genotype

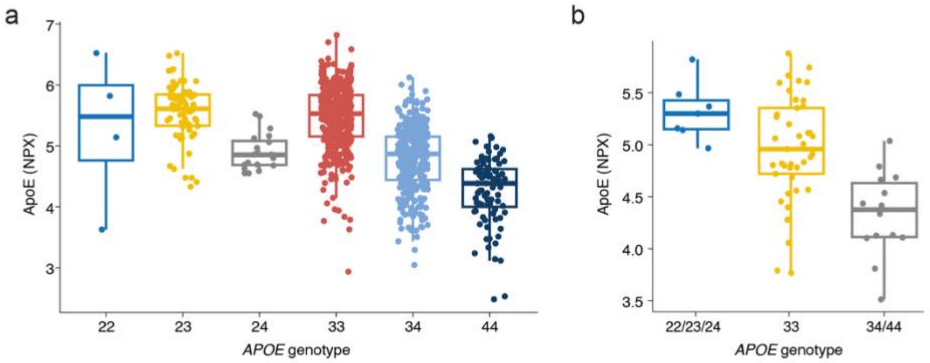

Legend: Dot plots showing the CSF ApoE levels across the different *APOE* genotypes in the whole sample (n=877) in **a** and only in individuals below 50 years old (n=62) in **b.** In both cases we see lower ApoE levels in *APOE*4 carriers, such that levels in 33 > 34 > 44. In all box plots, the box limits represent the first and third quartile, the line depicts the median value and the whisker extends to 1.5 time the interquartile range.

Supplementary Figure 2. Differential protein abundance analyses adjusting for *APOE*4 genotype

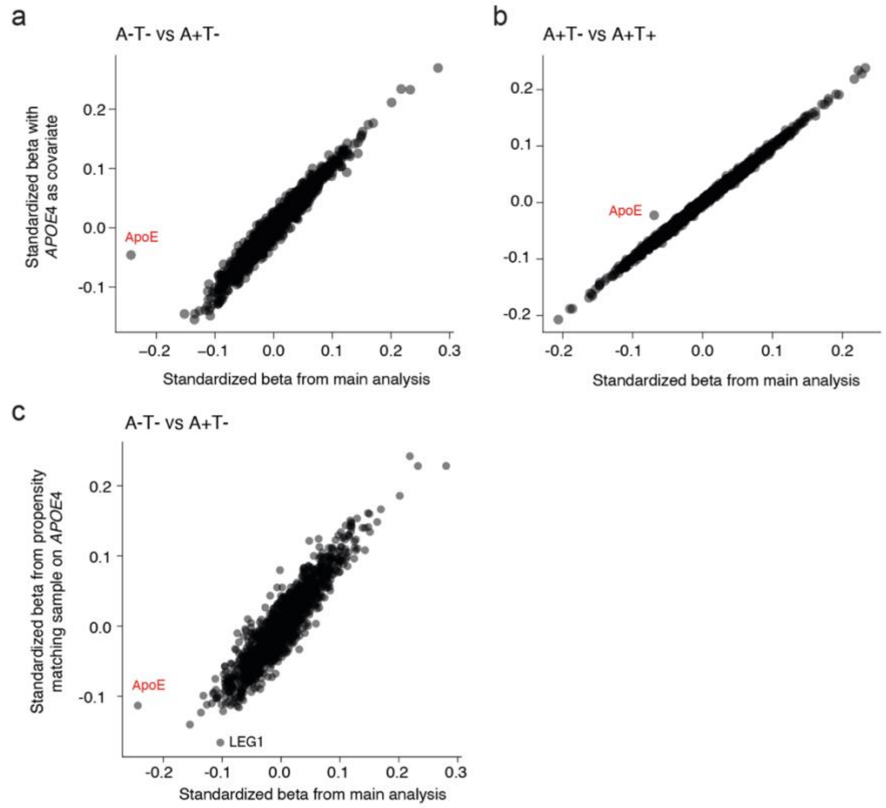

Legend: **a,** Comparison of the standardized betas from the main analyses assessing differential protein abundance between A+T- and A-T- in the x-axis vs. the standardized betas in analyses when further adding *APOE*4 genotype (having an e4 allele or not) as a covariate. **b,** Similar plot as in a, in the comparison between A+T+ and A+T-. **c,** Comparison of the standardized betas from the main analyses assessing differential protein abundance between A+T- and A-T- in the whole group in the x-axis vs. a subsample where the proportion of e4 carriers is matched in both groups (n=184, 40% *APOE*4 in A-T; n=184, 34% *APOE*4 in A+T-). Across all analyses, ApoE is the only protein confounded by the *APOE*4 genotype.

Supplementary Figure 3. DDC levels across diagnoses in the non-AD group

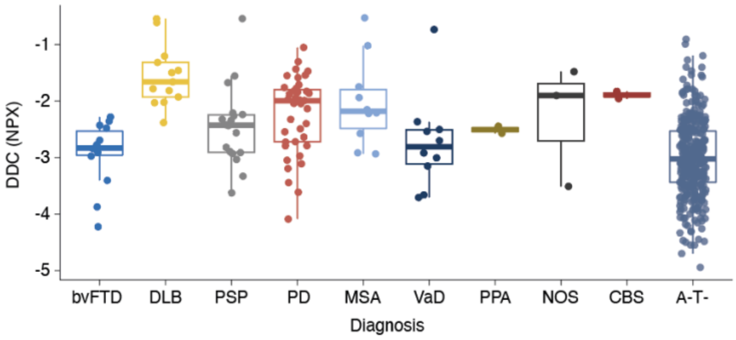

Legend: DDC (DOPA decarboxylase) levels across the different diagnoses in the group with non-AD neurodegenerative diseases (n=110) and in the A-T- group (n=352) as reference. The box limits represent the interquartile range and the line depicts the median value.
bvFTD= behavioral variant frontotemporal dementia; DLB= dementia with Lewy bodies; PSP= progressive supranuclear palsy; PD= Parkinson's disease; MSA= multiple system atrophy; VaD= vascular dementia; PPA= primary progressive aphasia; NOS= neurodegenerative disorder unspecified; CBS= corticobasal syndrome

Supplementary Figure 4. Aβ and tau markers mapped on the 2D representation of the pseudotime

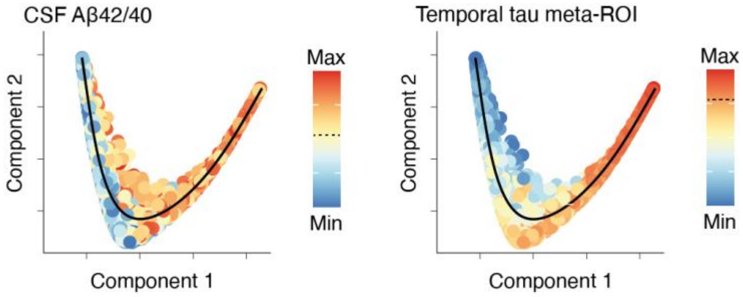

Legend: Biomarkers used to create the A (CSF Aβ42/40) and T (temporal meta-ROI tau PET uptake) groupings can be mapped on the 2D graph used to generate the pseudotime. Aβ abnormality could be seen around halfway along the progression, whereas abnormality on tau became evident in the last quarter of the pseudotime. The dashed line represents the threshold of positivity for the respective markers.

Supplementary Figure 5. Comparison of modules correspondence with previously published WGCNA modules

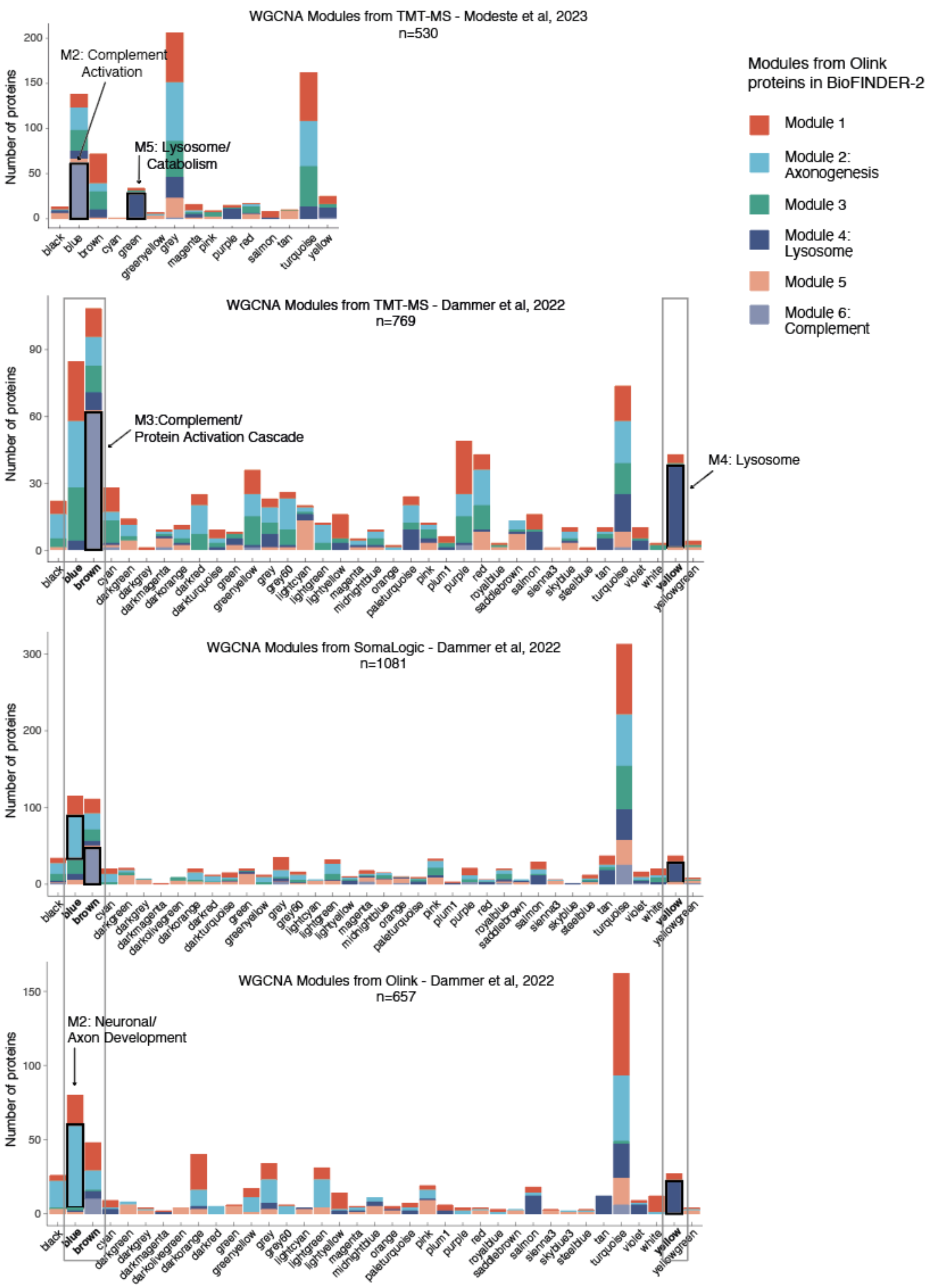

Legend: Olink protein distribution across previously identified modules derived from WGCNA from two published articles (Modeste et al., *Molecular Neurodegeneration*, Volume 18, Article number: 48, 2023 [top graph]; Dammer et al., *Alzheimer's Research & Therapy*, Volume 14, Article number: 174, 2022 [bottom three graphs]). Comparisons with Dammer et al. were split into three graphs, based on the three different technologies from which proteomic data was analyzed. The colors in the stacked barplots correspond to the Olink module derived from the current project. For each graph, the number of proteins overlapping between current Olink proteins and the dataset of comparison is reported in the

title. Overall, the WGCNA modules identified by Dammer et al. or Modeste et al. represent a mix of proteins from various Olink modules, but there were still a few modules that showed high consistency, highlighted with rectangles in the figure.
Our Module 6, related to complement activation, nicely mapped onto similar modules in both previous papers: M2 (blue): Complement activation in Modeste et al.; M3 (brown): Complement/Protein activation cascade in Dammer et al. Our Module 4, related to lysosomes and vacuoles, showed high correspondence with M5 (green): Lysosome/Catabolism in Modeste et al. and M4 (yellow): Lysosome in Dammer et al. Our Module 2, which contained many proteins related to axonogenesis, was highly distributed in M2 (blue): Neuronal/Axon development in Dammer et al., especially in the Olink proteins from the latter study.
TMT-MS= tandem mass tag mass spectrometry; WGCNA= weighted-gene co-expression analysis network

Supplementary Table 9. Olink panel statistics

**Olink panel Explore 3072**	**Proteins with > 70%** samples above limit of detection
Cardiometabolic	246 / 369 (67%)
Cardiometabolic II	130 / 367 (35%)
Inflammation	185 / 368 (50%)
Inflammation II	187 / 369 (51%)
Neurology	199 / 367 (54%)
Neurology II	98 / 367 (27%)
Oncology	180 / 368 (49%)
Oncology II	106 / 368 (29%)
**Total**	**1331/2943 (45%)**

Panel statistics of proteins for which > 70% of samples are above limit of detection (missing frequency < 30%)
