# Supplementary material for: Proteomic changes in Alzheimer’s disease associated with progressive Aβ plaque and tau tangle pathologies
Source: Nat Neurosci. 2024 Aug 26;27(10):1880–91. doi: 10.1038/s41593-024-01737-w (PMC11452344; doi:10.1038/s41593-024-01737-w)
Supplement: Supplementary file 2 — Reporting Summary [file 41593_2024_1737_MOESM2_ESM.pdf]

nature portfolio

Corresponding author(s): Alexa Pichet Binette & Oskar Hansson

Last updated by author(s): May 27, 2024

Reporting Summary

Nature Portfolio wishes to improve the reproducibility of the work that we publish. This form provides structure for consistency and transparency in reporting. For further information on Nature Portfolio policies, see our Editorial Policies and the Editorial Policy Checklist.

Statistics

For all statistical analyses, confirm that the following items are present in the figure legend, table legend, main text, or Methods section.

n/a	Confirmed	
☐	☒	The exact sample size (*n*) for each experimental group/condition, given as a discrete number and unit of measurement
☐	☒	A statement on whether measurements were taken from distinct samples or whether the same sample was measured repeatedly
☐	☒	The statistical test(s) used AND whether they are one- or two-sided<br>*Only common tests should be described solely by name; describe more complex techniques in the Methods section.*
☐	☒	A description of all covariates tested
☐	☒	A description of any assumptions or corrections, such as tests of normality and adjustment for multiple comparisons
☐	☒	A full description of the statistical parameters including central tendency (e.g. means) or other basic estimates (e.g. regression coefficient) AND variation (e.g. standard deviation) or associated estimates of uncertainty (e.g. confidence intervals)
☐	☒	For null hypothesis testing, the test statistic (e.g. *F*, *t*, *r*) with confidence intervals, effect sizes, degrees of freedom and *P* value noted<br>*Give P values as exact values whenever suitable.*
☒	☐	For Bayesian analysis, information on the choice of priors and Markov chain Monte Carlo settings
☒	☐	For hierarchical and complex designs, identification of the appropriate level for tests and full reporting of outcomes
☐	☒	Estimates of effect sizes (e.g. Cohen's *d*, Pearson's *r*), indicating how they were calculated

Our web collection on statistics for biologists contains articles on many of the points above.

Software and code

Policy information about availability of computer code

Data collection	No software was used.
Data analysis	R version 4.2.1 and python version 3.9.2 was used for all statistical analyses.<br>The main packages used were ggplot2 v3.4.4, stats v.4.3.2, lme4 v1.1-35.1, Seurat v4.3.0, EWCE v1.6.0. Brain renderings were created using the Connectome WorkBench software v.1.5.0. For neuroimaging processing, FreeSurfer v6.0 and ANTs v2.3.1 were used. Please see https://github.com/alexapichet/NatureNeuro_2024_proteomics for code used in this manuscript.

For manuscripts utilizing custom algorithms or software that are central to the research but not yet described in published literature, software must be made available to editors and reviewers. We strongly encourage code deposition in a community repository (e.g. GitHub). See the Nature Portfolio guidelines for submitting code & software for further information.

Data

Policy information about availability of data

All manuscripts must include a data availability statement. This statement should provide the following information, where applicable:

- Accession codes, unique identifiers, or web links for publicly available datasets
- A description of any restrictions on data availability
- For clinical datasets or third party data, please ensure that the statement adheres to our policy

BioFINDER data are available from the principal investigator (OH), pseudonymized data will be shared by request from a qualified academic investigator for the sole

purpose of replicating procedures and results presented in the article and as long as data transfer is in agreement with EU legislation on the general data protection regulation and decisions by the Ethical Review Board of Sweden and Region Skåne, which should be regulated in a data transfer agreement. ADNI data used in this manuscript are publicly available from the ADNI database (adni.loni.usc.edu) upon registration and compliance with the data use agreement. Single-nuclei RNAseq data from ROSMAP is available at https://www.synapse.org/#!Synapse:syn52293433 upon data use agreement. Single-nuclei RNAseq from the Allen Brain Institute is openly available at https://portal.brain-map.org/atlases-and-data/rnaseq. Summary statistics from all analyses are provided in Source Data.

Research involving human participants, their data, or biological material

Policy information about studies with human participants or human data. See also policy information about sex, gender (identity/presentation), and sexual orientation and race, ethnicity and racism.

Reporting on sex and gender	Sex was self-reported. Sex was included as a covariate in all analyses.
Reporting on race, ethnicity, or other socially relevant groupings	Race and ethnicity were not included as confounding factors in this manuscript. The BioFINDER-2 cohort is relatively homogeneous with a vast majority of White individuals from south of Sweden.
Population characteristics	Detailed information is given in Table 1 and in Extended Data Table 1.
Recruitment	In BioFINDER-2, the sample consisted of patients that had been referred to participting memory clinics (mostly from primary care) and most cognitively unimpaired participants were recruited from the general population in the south of Sweden. Informed consent was obtained from all participants and they were compensated for each study visit they completed.
Ethics oversight	Ethical approval was given by the Regional Ethical Committee of Lund University.

Note that full information on the approval of the study protocol must also be provided in the manuscript.

Field-specific reporting

Please select the one below that is the best fit for your research. If you are not sure, read the appropriate sections before making your selection.

☒ Life sciences ☐ Behavioural & social sciences ☐ Ecological, evolutionary & environmental sciences

For a reference copy of the document with all sections, see nature.com/documents/nr-reporting-summary-flat.pdf

Life sciences study design

All studies must disclose on these points even when the disclosure is negative.

Sample size	We did not a priori perform a sample size calculation for this study. We selected the largest sample size available with participants who had CSF proteomics as well as measures of amyloid and tau pathology, for a total of 879 participants. This sample size is among the largest available with in vivo CSF proteomics and deep-phenotyping.
Data exclusions	Proteins for which more than 70% of participants had measurements below the limit of detection were excluded and not considered for analyses. This exclusion criteria resulted in 1331 proteins analyzed.
Replication	We used two independent cohorts to validate the main proteomics hits in CSF in relation to amyloid pathology (measures of tau pathology were not available in these cohorts): BioFINDER-1 with Olink proteomics and ADNI with SomaLogic proteomics. Analyses were performed once and independently, on the overlapping set of proteins available in BioFINDER-2 and the replication cohorts.
Randomization	Based on amyloid and tau status we generated different biomarker groups: A-T-, A+T- and A+T+ as well as a group of amyloid-negative with non-AD neurodegenerative diseases.
Blinding	Proteomic measurements were performed blinded to any demographics or clinical characteristics.

Reporting for specific materials, systems and methods

We require information from authors about some types of materials, experimental systems and methods used in many studies. Here, indicate whether each material, system or method listed is relevant to your study. If you are not sure if a list item applies to your research, read the appropriate section before selecting a response.

Materials & experimental systems

n/a	Involved in the study
☐	☒ Antibodies
☒	☐ Eukaryotic cell lines
☒	☐ Palaeontology and archaeology
☒	☐ Animals and other organisms
☐	☒ Clinical data
☒	☐ Dual use research of concern
☒	☐ Plants

Methods

n/a	Involved in the study
☒	☐ ChIP-seq
☒	☐ Flow cytometry
☒	☐ MRI-based neuroimaging

Antibodies

Antibodies used	Details about antibody-based proteomic technology is provided by Olink, with all details found here: https://www.olink.com/content/uploads/2021/09/olink-white-paper-pea-a-high-multiplex-immunoassay-technology-with-qpcr-or-ngs-readout-v1.0.pdf<br><br>Tau1 (generated by Nicholas Kanahan) and HJ series (HJ8.5, HJ8.7 and HJ34.8) antibodies (generated by Dr. David Holtzman) were used to measure p-tau.<br><br>For immunofluorescent staining, we used SMOC1 (WH0064093M3 clone 8F10, Merck), p-tau 231 (ab151559, Abcam), Methoxy-X04 (4920, Tocris Biotechne), Goat anti-rabbit 488 1:200 (Invitrogen, A11008) and goat anti-mouse 549 1:200 (Invitrogen, A11029).
Validation	Extensive validation has been conducted by Olink, with all details of the Explore 3072 available here: https://olink.com/content/uploads/2022/10/olink-explore-validation-data.pdf<br><br>Tau1, HJ8.5 and 8.7 were validated in the following studies:<br>Barthélemy NR et al. Site-Specific Cerebrospinal Fluid Tau Hyperphosphorylation in Response to Alzheimer's Disease Brain Pathology: Not All Tau Phospho-Sites are Hyperphosphorylated. J Alzheimers Dis 2022;85(1):415-429.<br>Sato C et al. Tau Kinetics in Neurons and the Human Central Nervous System. Neuron 2018 Mar 21;97(6):1284-1298.e7

Clinical data

Policy information about clinical studies

All manuscripts should comply with the ICMJE guidelines for publication of clinical research and a completed CONSORT checklist must be included with all submissions.

Clinical trial registration	BioFINDER-2: NCT03174938; BioFINDER-1: NCT01208675; ADNI: NCT00106899
Study protocol	Please see www.biofinder.se for BioFINDER studies. Please see www.adni-info.org or ida.loni.usc.edu for ADNI.
Data collection	BioFINDER-2 participants include a mix of population-based and memory clinic-based studies in Lund and Malmö, in Sweden and all imaging data was acquired at Skane University Hospital between April 2017 and December 2022.<br>BioFINDER-1 participants include a mix of population-based and memory clinic-based studies in Lund and Malmö, in Sweden. All participants were enrolled and data acquired between July 2009 and March 2015.<br>ADNI is a multi-site study that is population-based, but mostly pools from academic centers and excludes for many comorbidities. ADNI data included in this study were all acquired between January 2005 and January 2013.<br>In all cohorts, informed consent was obtained from all participants and they were compensated for their participation.
Outcomes	The predefined primary outcome measures were the differentially expressed proteins between the different A/T groups.<br>After having identified the differentially expressed proteins, we then evaluated different outcomes based on 1) functional enrichment analyses from Gene Ontology databases, 2) cell-type enrichment analyses from single-cell transcriptomics data, 3) associations to amyloid and tau measured with PET cross-sectionally and longitudinally. Secondary outcomes 1 and 2 were chosen given that they are state-of-the-art outcomes in omics studies and 3 as it is the gold-standard for measuring AD pathology in vivo.

Plants

Seed stocks	*Report on the source of all seed stocks or other plant material used. If applicable, state the seed stock centre and catalogue number. If plant specimens were collected from the field, describe the collection location, date and sampling procedures.*
Novel plant genotypes	*Describe the methods by which all novel plant genotypes were produced. This includes those generated by transgenic approaches, gene editing, chemical/radiation-based mutagenesis and hybridization. For transgenic lines, describe the transformation method, the number of independent lines analyzed and the generation upon which experiments were performed. For gene-edited lines, describe the editor used, the endogenous sequence targeted for editing, the targeting guide RNA sequence (if applicable) and how the editor was applied.*
Authentication	*Describe any authentication procedures for each seed stock used or novel genotype generated. Describe any experiments used to assess the effect of a mutation and, where applicable, how potential secondary effects (e.g. second site T-DNA insertions, mosiacism, off-target gene editing) were examined.*
